# Blood-derived lysophospholipid sustains hepatic phospholipids and fat storage necessary for hepatoprotection in overnutrition

**DOI:** 10.1172/JCI171267

**Published:** 2023-09-01

**Authors:** Cheen Fei Chin, Dwight L.A. Galam, Liang Gao, Bryan C. Tan, Bernice H. Wong, Geok-Lin Chua, Randy Y.J. Loke, Yen Ching Lim, Markus R. Wenk, Miao-Shan Lim, Wei-Qiang Leow, George B.B. Goh, Federico Torta, David L. Silver

**Affiliations:** 1 Signature Research Program in Cardiovascular and Metabolic Disorders, Duke-NUS Medical School, Singapore.; 2 Singapore Lipidomics Incubator, Life Sciences Institute and; 3 Precision Medicine Translational Research Programme and Department of Biochemistry, Yong Loo Lin School of Medicine, National University of Singapore, Singapore.; 4 Department of Gastroenterology and Hepatology, Singapore General Hospital, Singapore.; 5 Department of Anatomical Pathology, Singapore General Hospital, and; 6 Medicine Academic Clinical Program, Duke-NUS Medical School, Singapore.

**Keywords:** Hepatology, Metabolism, Homeostasis, Mouse models, Transport

## Abstract

The liver has a high demand for phosphatidylcholine (PC), particularly in overnutrition, where reduced phospholipid levels have been implicated in the development of nonalcoholic fatty liver disease (NAFLD). Whether other pathways exist in addition to de novo PC synthesis that contribute to hepatic PC pools remains unknown. Here, we identified the lysophosphatidylcholine (LPC) transporter major facilitator superfamily domain containing 2A (Mfsd2a) as critical for maintaining hepatic phospholipid pools. Hepatic Mfsd2a expression was induced in patients having NAFLD and in mice in response to dietary fat via glucocorticoid receptor action. Mfsd2a liver-specific deficiency in mice (*L2aKO*) led to a robust nonalcoholic steatohepatitis–like (NASH-like) phenotype within just 2 weeks of dietary fat challenge associated with reduced hepatic phospholipids containing linoleic acid. Reducing dietary choline intake in *L2aKO* mice exacerbated liver pathology and deficiency of liver phospholipids containing polyunsaturated fatty acids (PUFAs). Treating hepatocytes with LPCs containing oleate and linoleate, two abundant blood-derived LPCs, specifically induced lipid droplet biogenesis and contributed to phospholipid pools, while LPC containing the omega-3 fatty acid docosahexaenoic acid (DHA) promoted lipid droplet formation and suppressed lipogenesis. This study revealed that PUFA-containing LPCs drive hepatic lipid droplet formation, suppress lipogenesis, and sustain hepatic phospholipid pools — processes that are critical for protecting the liver from excess dietary fat.

## Graphical abstract

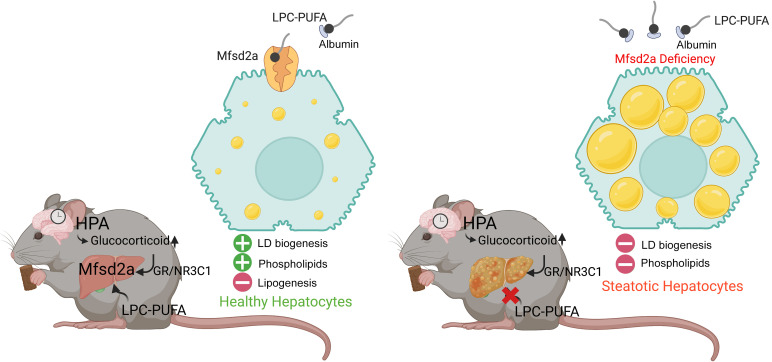

## Introduction

Hepatic accumulation of dietary lipids is caused by excessive uptake and impairment in lipid disposal. There are 4 major routes whereby liver maintains the balance of hepatic lipids, namely, through uptake of circulating lipids as unesterified fatty acids and lipoproteins, de novo lipogenesis (DNL), fatty acid oxidation, and lipid export via VLDL secretion (reviewed in ref. 1). Alternatively, the liver can buffer against lipotoxicity by incorporating excessive unesterified fatty acids into triglycerides (TGs) and store them in cytosolic lipid droplets (LDs) (2). The majority of patients with nonalcoholic fatty liver disease (NAFLD) present with macrosteatosis, characterized by the presence of few, large LDs in hepatocytes (3). The disposal of lipids through VLDL export, storage through formation of small LDs, and bile acid secretion require a constant supply of phospholipids (reviewed in refs. 4, 5). Indeed, hepatocyte phospholipid pools are rapidly turned over and replaced by new phospholipids (6), consistent with the liver having a high demand for phosphatidylcholine (PC) to maintain these physiological functions. Interestingly, mass spectrometry–based lipidomic analysis of human liver biopsies and livers from rodent models of NAFLD revealed aberrant alteration in the phospholipid pools, suggesting that dysregulation of phospholipid metabolism is associated with the development of NAFLD (7, 8). In fact, PC depletion in liver through use of choline- or choline and methionine–deficient diets is one of the most classically used methods for inducing NAFLD in rodent models (9–11), supporting the conclusion that hepatic PC is particularly limiting in overnutrition with dietary fat.

In hepatocytes, PC containing the essential fatty acid linoleate (FA-18:2, denotes 18 carbons: 2 double bonds) is one of the most abundant PC species in hepatic membranes and in the phospholipid shell of LDs (12–14). The origin of hepatic PC is derived from 2 distinct de novo synthesis routes, namely, the CDP/choline pathway (Kennedy pathway) and the phosphatidylethanolamine (PE) *N*-methyltransferase (PEMT) pathway. The CDP/choline pathway uses choline as an initial substrate for PC synthesis (15), accounting for approximately 70% of hepatic PC production (16). In contrast, PEMT catalyzes the conversion of PE to PC via 3 methylation reactions using *S*-adenosyl methionine (SAM) as the methyl donor (17), accounting for the remaining 30% of the PC pool in the liver under conditions of normal dietary fat intake (16). Whether other physiological pathways exist by which the liver obtains PC, particularly in overnutrition, is not known.

We identified major facilitator superfamily domain containing 2A (*Mfsd2A*) as a sodium-dependent lysophosphatidylcholine (LPC) symporter that is highly expressed at the blood-brain barrier (BBB) endothelium and blood-retinal barriers (18–20). Blood-derived LPCs are transported on albumin and are of limited diversity, with LPC-18:1 and LPC-18:2 being 2 of the more abundant LPC species found in rodent and human blood (21). Mfsd2a has high specificity for transporting LPCs with mono- and polyunsaturated fatty acids and constitutes the major pathway by which the brain and eye obtains the omega-3 fatty acid docosahexaenoic acid (DHA) (LPC-22:6) (18, 19). *Mfsd2a-*KO mice exhibited severe microcephaly with reduced neuron arborization and increased de novo fatty acid biosynthesis through upregulation of SREBP1 activity that correlated with compensatory changes in phospholipid composition, indicating that LPC uptake into brain is critical for regulating phospholipid pools (18, 19, 22). Similarly to mice deficient in Mfsd2a, humans with homozygous loss-of-function mutations in *Mfsd2a* exhibited severe microcephaly and intellectual disability, categorized as primary autosomal microcephaly 15 (23–26), indicating that Mfsd2a-mediated LPC transport is essential for human brain development.

In contrast to the constitutive expression of Mfsd2a in brain and eyes, liver Mfsd2a is expressed in a spatially and temporally regulated fashion. Hepatic Mfsd2a is exclusively expressed in periportal hepatocytes (27), with expression found to be extremely low in fed conditions and robustly induced by fasting in a PPARα and glucagon receptor–signaling dependent manner (28, 29). In addition to being induced by fasting, Mfsd2a liver expression is also under superchiasmatic nucleus (SCN) clock-dependent circadian control (30). Despite some understanding of the transcription factors that govern the tight regulation of Mfsd2a expression in response to fasting, the function of blood-derived LPC transport by Mfsd2a in the liver remains enigmatic. In this study, we demonstrate that blood-derived LPC transport by Mfsd2a protects the liver from pathologies consequential to overnutrition by stimulating LD formation and supplying the liver with phospholipids.

## Results

### Mfsd2a is an early response gene during overnutrition.

It has been previously shown that Mfsd2a is expressed specifically in periportal hepatocytes (27). Consistent with that report, using a Mfsd2a lineage tracing mouse model, *Mfsd2a-CreERT2 Rosa26-tdtomato* (31), we observed tdtomato^+^ hepatocytes colocalized specifically with E-cadherin, a periportal hepatocyte marker, but not with the pericentral hepatocyte marker glutamine synthetase (Supplemental Figure 1A; supplemental material available online with this article; https://doi.org/10.1172/JCI171267DS1). This finding is corroborated by analysis of publicly available mouse and human liver single-cell RNA-Seq data sets showing *Mfsd2a* mRNA is enriched in hepatocytes surrounding the portal triad (zone 1) of mouse and human liver (32, 33). Given that Mfsd2a transport function is sodium dependent, we anticipated that it would be expressed on the basolateral (blood facing) side of hepatocytes. Unfortunately, our Mfsd2a antibody did not detect endogenous Mfsd2a in mouse liver sections, suggesting that the C-terminal epitope to which the antibody was generated is blocked. In contrast, Mfsd2a was detectable at the basolateral membrane when overexpressed in mouse liver using a doxycycline-inducible transgenic mouse line (Supplemental Figure 1B). The localization of Mfsd2a to the basolateral membrane is consistent with Mfsd2a being a sodium-dependent transporter.

**Figure 1:**
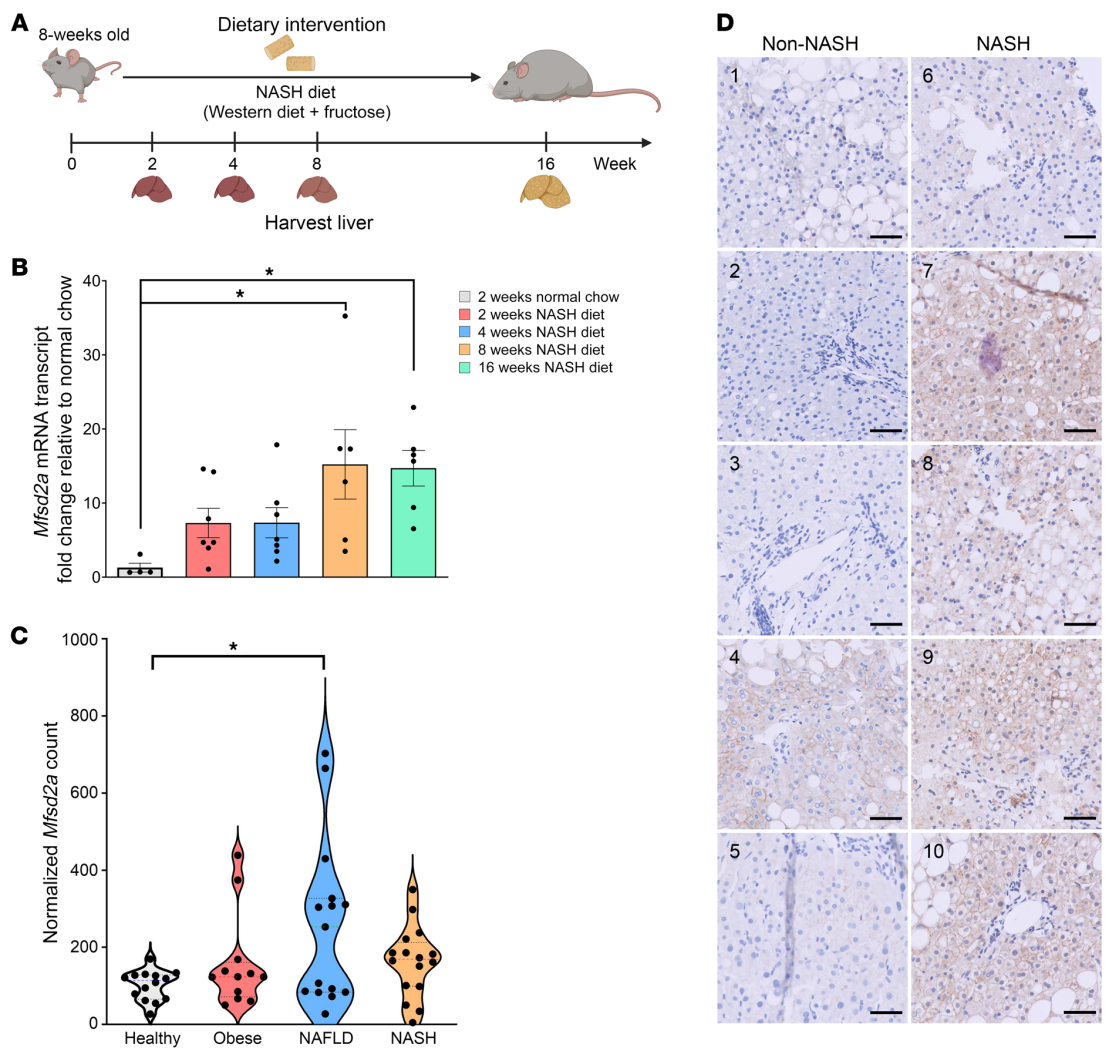
Hepatic *Mfsd2a* is an early response gene and is upregulated with increasing NAFLD severity in patients. (**A**) Feeding regimen for NASH diet–induced fatty liver in WT mice. (**B**) Levels of hepatic *Mfsd2a* expression in WT mice fed a NASH diet relative to normal chow–fed mice over time (2 weeks, *n* = 7; 4 weeks, *n* = 7; 8 weeks, *n* = 6; 16 weeks, *n* = 6; normal chow fed controls, *n* = 4). *Mfsd2a* mRNA expression was normalized to β-actin. Data are represented as the mean ratio of normalized *Mfsd2a* relative to *Mfsd2a* expression in normal chow–fed mice ± SEM. *P < 0.05; **P < 0.01, 1-way ANOVA with Dunnett’s test. (**C**) Hepatic *Mfsd2a* expression levels in individuals of normal weight (*n* = 14), obese individuals (*n* = 12), and individuals with NAFLD (*n* = 15) and NASH (*n* = 16). Normalized *Mfsd2a* expression was analyzed from RNA-Seq data set GSE126848. Data are expressed as normalized count. *P < 0.05, 1-way ANOVA with Tukey’s test. (**D**) Immunostaining of Mfsd2a in liver sections from patients without NASH (*n* = 5) and with NASH (*n* = 5). Numbers on each image correspond to patient identification numbers in Supplemental Table 1. Scale bars: 50 μm.

Given that Mfsd2a is expressed specifically in periportal hepatocytes, a zone that is the first to receive dietary lipids, we asked whether Mfsd2a expression is altered in response to overnutrition stress. To test this idea, *Mfsd2a* expression was quantified in livers at 2, 4, 8, and 16 weeks after nonalcoholic steatohepatitis (NASH) diet feeding (Figure 1, A and B). Interestingly, *Mfsd2a* mRNA expression was induced as early as 2 weeks and significantly elevated at 8 and 16 weeks after NASH diet feeding (Figure 1B), suggesting that LPC transport by Mfsd2a plays a physiological role in the early response to high-fat diet (HFD) challenge.

### Hepatic Mfsd2a expression is upregulated with increasing NAFLD severity in patients.

We examined a publicly available liver RNA-Seq data set from a cohort of healthy normal weight and obese individuals as well as NAFLD and NASH patients (34). Consistent with our mouse data, liver *Mfsd2a* mRNA expression was significantly higher in patients with NAFLD compared with healthy normal-weight individuals (Figure 1C). To provide an independent confirmation of these mRNA findings, we recruited 5 patients with NASH and 5 patients without NASH from Singapore General Hospital (Supplemental Table 1) and examined Mfsd2a protein expression using IHC in liver biopsies obtained in these patients. Mfsd2a expression was highly elevated in NASH patients, evident from the intense plasma membrane staining of Mfsd2a in periportal hepatocytes that was absent in the non-NASH patients, with the exception of one patient who had moderate steatosis and inflammation (Figure 1D). This result suggests that upregulation of hepatic Mfsd2a might also be an early event in the development of NAFLD, similar to its upregulation in response to NASH diet challenge in mice.

### Liver-specific Mfsd2a deficiency resulted in the development of severe steatohepatitis and fibrosis.

**Figure 2:**
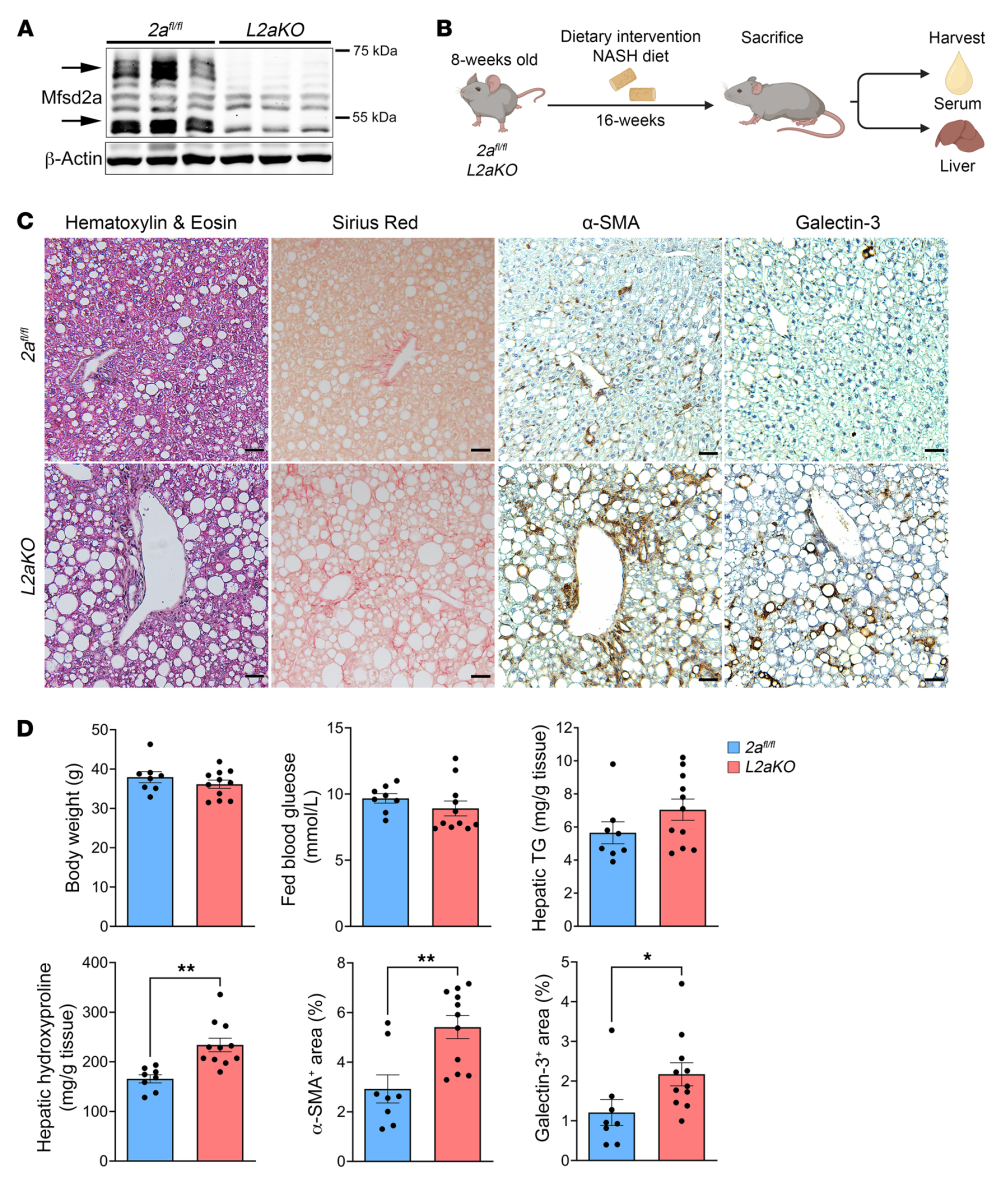
Liver-specific *Mfsd2a* deficiency resulted in development of severe steatohepatitis and fibrosis. (**A**) Immunoblot showing the expression of Mfsd2a in *2a^fl/fl^* and its absence in *L2aKO* mouse liver (*n* = 6 per genotype). β-Actin was used as a loading control. Arrows indicate Mfsd2a, which appears in the liver as 2 distinct molecular weight species due to glycosylation (45). (**B**) Feeding regimen for mice on NASH diet. (**C**) Histological analysis was performed on liver sections following 16 weeks of NASH diet feeding using H&E, Sirius red, α-SMA, and galectin-3. Scale bars: 50 μm. (**D**) Morphometry analysis quantified the percentages of area that were positively stained for each of the indicated markers (*2a^fl/fl^*, *n* = 8; *L2aKO*, *n* = 11). Data are represented as means ± SEM. *P < 0.05; **P < 0.01, 2-tailed Welch’s *t* test.

Global *Mfsd2a*-KO mice are small and have severe microcephaly (22). In addition, these mice are lean in part due to increased energy expenditure and increased fatty acid β-oxidation in brown adipose tissue (28). These major phenotypes are confounders in delimiting a function of Mfsd2a in liver. To circumvent this issue, we generated a liver-specific *Mfsd2a*-KO line (*L2aKO*) by using a *Mfsd2a* mouse line with floxed alleles (*2a^fl/fl^*) (19) crossed to an albumin promoter–driven Cre recombinase (*Alb-Cre*) transgenic mouse line. The deletion of *Mfsd2a* in hepatocytes was highly efficient, as evidenced from the absence of Mfsd2a protein expression in whole liver lysates (Figure 2A).

To determine whether Mfsd2a is important for the hepatic response to HFD feeding, we challenged both 8-week-old adult *2a^fl/fl^* and *L2aKO* mice with a NASH diet for 16 weeks (Figure 2B). Body weight and fed blood glucose levels did not differ between *2a^fl/fl^* and *L2aKO* (Figure 2D). Examination of livers of mice fed for 16 weeks with a NASH diet revealed that *L2aKO* livers had ballooning hepatocytes and severe macrosteatosis at the periportal region that were uncommon in *2a^fl/fl^* livers (Figure 2C). This pathology in periportal hepatocytes is consistent with Mfsd2a expression being specific to this zone (Supplemental Figure 1). We also observed a significant increase in percentage area of galectin-3–positive (inflammatory cell marker) cells relative to *2a^fl/fl^* controls, consistent with severe steatohepatitis (Figure 2, C and D). The presence of severe steatohepatitis in *L2aKO* mice at the 16-week time point was associated with elevation of the activated hepatic stellate cell (HSC) profibrogenic marker α-SMA and significantly increased collagen deposition relative to *2a^fl/fl^* controls, as assessed by Sirius red staining and quantification of total hydroxyproline (Figure 2, C and D).

### Diet-induced expression of Mfsd2a is controlled by the glucocorticoid receptor.

A previous study demonstrated that circadian control of *Mfsd2a* is under brain clock control and not under hepatic clock control (30). We have confirmed that *Mfsd2a* mRNA transcripts were low at zeitgeber 0 (ZT0) (lights on) and peaked at ZT12 (lights off), coinciding with the onset of mealtime that returned to near baseline within 4 hours (ZT16) (Supplemental Figure 2A). Mfsd2a protein followed a circadian pattern similar to that of *Mfsd2a* mRNA (Supplemental Figure 2B). These data suggest the existence of a circulating factor that regulates *Mfsd2a* in a circadian fashion.

**Figure 3:**
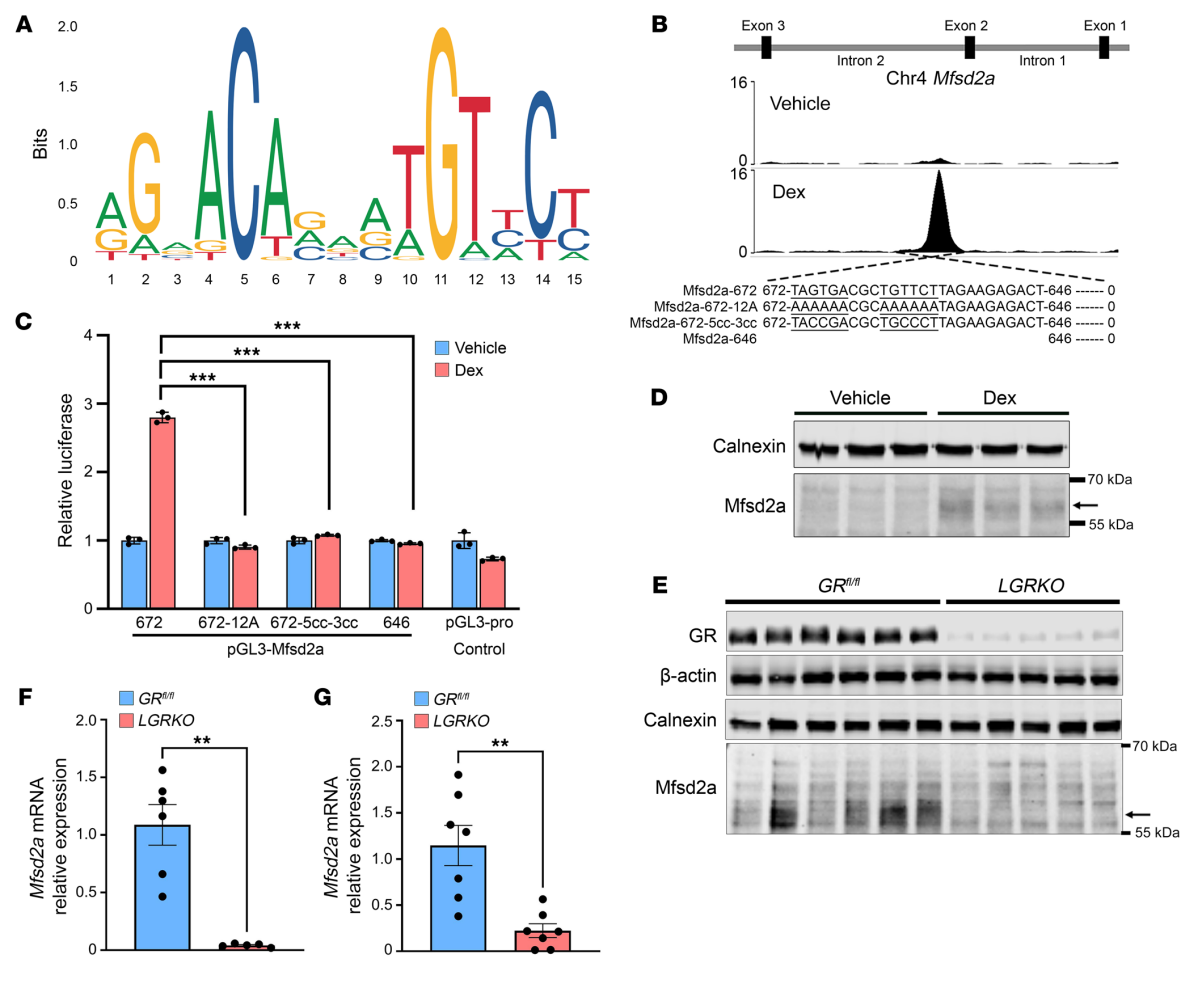
*Mfsd2a* is regulated by GR during overnutrition stress. (**A**) GRE consensus motifs. (**B**) Chip-Seq profile showing GR occupancy at intron 2 in Dex- or vehicle-injected mice. (**C**) Luciferase reporter assay defines the GRE in *Mfsd2a* intron 2. HeLa cells were transfected with the indicated deletions or point mutation constructs of *Mfsd2a* intron 2 fused upstream of luciferase. Data are expressed as fold change of luciferase activity in 100 nM Dex-treated relative to vehicle-treated control cells, means ± SEM. (**D**) Immunoblot analysis showing the protein expression of Mfsd2a, indicated by the arrow, in 5 mg/kg Dex-injected (*n* = 4) or vehicle-injected (*n* = 4). GAPDH was used as a loading control. (**E**) *Mfsd2a* protein expression in *GR^fl/fl^* (*n* = 6) and *LGRKO* mice (*n* = 5) fed a normal chow diet at ZT12 (fasted for 12 hours). (**F**) *Mfsd2a* mRNA expression in *GR^fl/fl^* (*n* = 6) and *LGRKO* mice (*n* = 5) fed a normal chow diet at ZT12 (fasted for 12 hours). (**G**) *Mfsd2a* mRNA expression in *GR^fl/fl^* and *LGRKO* mice (*n* = 7 per genotype) fed a NASH diet for 2 weeks. Data are represented as means ± SEM. **P < 0.001; ***P < 0.01, 2-tailed Welch’s *t* test.

Mining a publicly available data set for NR3C1 binding sites in mouse liver (35) revealed that NR3C1 (glucocorticoid receptor [GR]) binds to intron 2 of the *Mfsd2a* gene in mouse liver treated with the GR agonist dexamethasone (Dex) (Figure 3B). Glucocorticoid response element (GRE) motif analysis using JASPAR 2022 (36) (Figure 3A) revealed 14 putative GRE-binding sites at this region. To identify the functional GRE sites, deletions of these putative GREs was generated and tested for GR activity using a luciferase reporter system in HeLa cells. A 672 bp full-length sequence flanking the GR peak in intron 2 showed robust luciferase activity in the presence of Dex (Figure 3C). Truncation of the GRE motif to 646 bp (646-GRE) completely abolished the Dex-induced luciferase activity (Figure 3C). Consistently, the Dex-induced luciferase activity was lost in 672-GRE having half-sites mutated to 12 adenines (672-12A) or having minimal half-site mutations to cytosine (672-5cc-3cc), confirming that the defined 672 bp of intron 2 is the site required for GR transactivation of Mfsd2a expression (Figure 3C). Injection of Dex into mice resulted in an induction of hepatic Mfsd2a protein (Figure 3D), consistent with *Mfsd2a* being a direct target of GR. Importantly, we found in analyzing a Cistrome data set derived from BSEA-2B cells (37), a human lung epithelial-like cell line, treated with Dex that this GRE in mouse intron 2 is conserved with a GR peak in intron 2 of human *Mfsd2a* (Supplemental Figure 3, A and B). To definitively prove that liver GR is responsible for circadian control of *Mfsd2a* expression, we generated a liver-specific *GR*-KO mouse (*LGRKO*) and assessed Mfsd2a expression at ZT12, the circadian peak of Mfsd2a expression (Supplemental Figure 2A). Importantly, peak *Mfsd2a* mRNA and protein expression were abolished in *LGRKO* mice at ZT12 (Figure 3, E and F).

Given that *Mfsd2a* is a direct GR target gene, we tested to determine whether NASH diet–induced elevation of hepatic Mfsd2a expression requires GR. Importantly, the NASH diet–induced expression of Mfsd2a was abolished in *LGRKO* mice (Figure 3G). These combined findings indicate that Mfsd2a expression peaks prior to mealtime under the control of GR and is physiologically important for the liver to respond to dietary lipid uptake.

### Mfsd2a deficiency results in diet-induced early onset steatohepatitis and reduced levels of linoleate- and DHA-containing phospholipids in liver.

**Figure 4:**
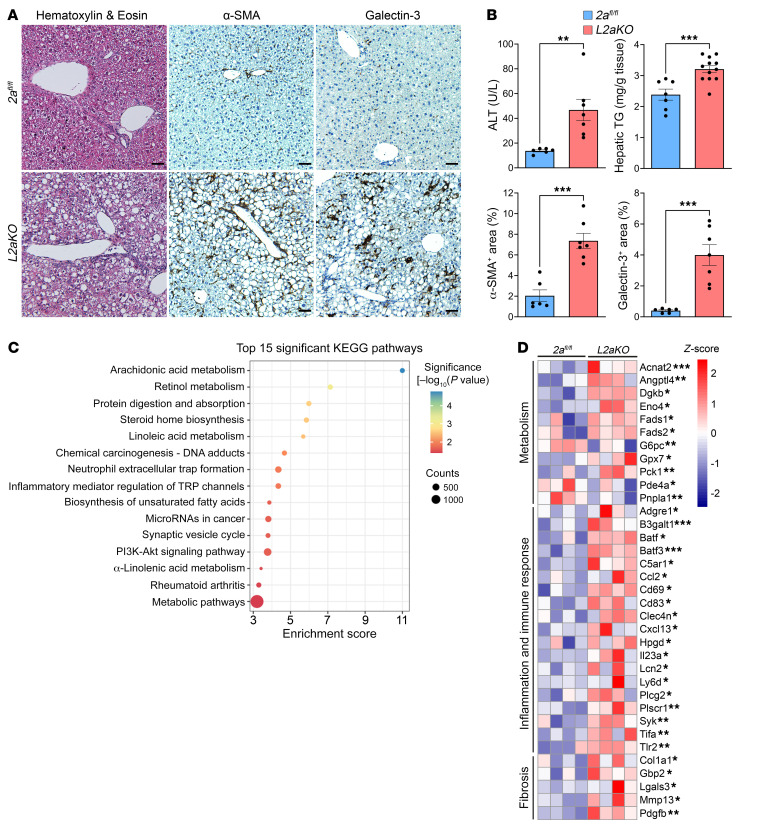
*Mfsd2a* deficiency results in diet-induced early onset steatohepatitis. (**A** and **B**) Adult *2a^fl/fl^* and *L2aKO* mice were fed with a NASH diet for 2 weeks (TG assay: *2a^fl/fl^*, *n* = 7, *L2aKO*, *n* = 12; IHC quantification: *2a^fl/fl^*, *n* = 6, *L2aKO*, *n* = 7). Histological analysis was performed on liver sections using H&E, α-SMA, and galectin-3. Scale bars: 50 μm. Morphometry analysis quantified the percentage areas that were positively stained for each of the indicated markers. Data are represented as means ± SEM. **P < 0.01; ***P < 0.001, 2-tailed Welch’s *t* test. (**C**) Hepatic RNA-Seq analysis in *2a^fl/fl^* and *L2aKO* mice fed a NASH diet for 2 weeks (*n* = 4 per genotype). Bubble plot shows the top 15 significant KEGG pathways for DEGs. (**D**) Heatmap representing the DEGs involved in relevant biological processes. Data are represented as *z* score of median ratio counts. *P < 0.05; **P < 0.01; ***P < 0.001.

Since Mfsd2a expression is induced via GR action as early as 2 weeks after NASH diet feeding (Figure 1B), we sought to determine whether Mfsd2a is important for protecting the liver during the early response to HFD challenge. *L2aKO* mice that were fed with a NASH diet for 2 weeks exhibited significantly elevated serum levels of alanine aminotransferase (ALT), indicative of liver damage (Figure 4B). Remarkably, *L2aKO* mice developed extensive periportal macrosteatosis, indicated by the accumulation of large LDs and elevated TGs, while livers of *2a^fl/fl^* mice were largely devoid of liver pathology (Figure 4A). Lobular inflammation was present specifically at the periportal region having severe macrosteatosis in the *L2aKO* mice (Figure 4A). IHC staining using the immune cell marker galectin-3 confirmed the presence of significantly increased levels of immune cells in the livers of *L2aKO* mice (Figure 4, A and B). Remarkably, α-SMA–positive cells were elevated in livers of *L2aKO* mice, indicative of early onset hepatic fibrosis (Figure 4, A and B).

As an independent assessment of liver pathology in *L2aKO* mice, global gene expression analysis using bulk RNA-Seq was performed on mice fed a NASH diet for 2 weeks. There were a total of 301 differentially expressed genes (DEGs) (183 upregulated and 118 downregulated genes, *P* < 0.05) in *L2aKO* relative to *2a^fl/fl^* livers. KEGG pathway analysis revealed that most DEGs were involved in lipid metabolism and inflammatory immune responses (Figure 4C). Consistent with the histopathology analysis, we observed an upregulation of genes in *L2aKO* involved in metabolism, inflammation and immune response, and fibrosis (Figure 4D). These data indicate that the Mfsd2a/LPC pathway is critical for protecting the liver from the development of diet-induced NAFLD.

**Figure 5:**
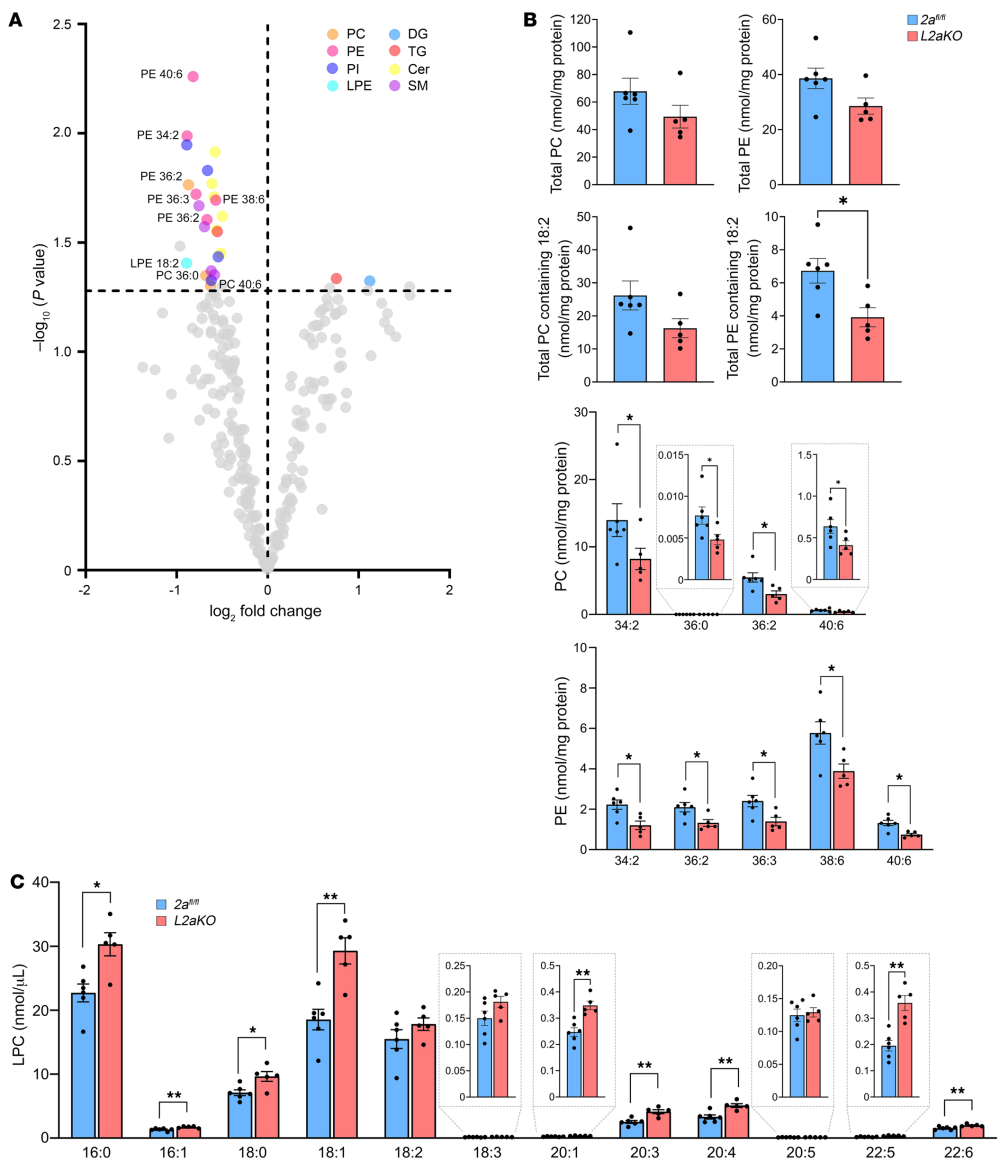
PC containing linoleate is reduced in NASH diet–fed *L2aKO* mice. (**A**) Lipidomic analysis of livers from *2a^fl/fl^* (*n* = 6) and *L2aKO* (*n* = 5) mice fed with NASH diet for 2 weeks. Volcano plot showing the significantly changed lipid species in *L2aKO* as compared with *2a^fl/fl^* mice (colored dots represent significant species and are located above dashed line, indicating a threshold of *P* < 0.05). (**B**) Graphs showing total PC, total PE, total PC containing 18:2, total PE containing 18:2, and significantly changed specific PC and PE species. (**C**) Significantly changed LPC species in serum from *2a^fl/fl^* and *L2aKO* mice fed with NASH diet for 2 weeks. Data are represented as means ± SEM. *P < 0.05; **P < 0.01, 2-tailed Welch’s *t* test.

We hypothesized that Mfsd2a could be important for supplying the liver with LPC for maintaining normal levels of PC during dietary lipid challenge. To initially test this concept, the liver lipid profile of *2a^fl/fl^* and *L2aKO* mice fed a NASH diet for 2 weeks was analyzed by mass spectrometry–based lipidomics. Targeted lipidomic analysis of 246 lipid species in 26 lipid classes revealed reductions in specific PE and PC species (Figure 5A), while the total PC and PE levels were similar between *2a^fl/fl^* and *L2aKO* livers (Figure 5B). A study from the Vance group determined that mice deficient in PEMT have a significantly reduced hepatic PC/PE ratio and early progression of steatosis to steatohepatitis when challenged with an HFD (38). In contrast, the PC/PE ratio remained unchanged between *L2aKO* mice and the *2a^fl/fl^* controls (Supplemental Figure 4). However, PC levels of linoleate (18:2), one of the most abundant fatty acids in hepatic PC and PC containing DHA (22:6), were significantly decreased across multiple PC species in *L2aKO* mice relative to *2a^fl/fl^* controls (Figure 5B). We observed a small but significant increase in plasma LPCs (carbon chain length ≥ 16) in *L2aKO* mice (Figure 5C), indicating that hepatic Mfsd2a contributes to regulating plasma LPC levels. Histological and lipidomic analysis of livers from *L2aKO* mice fed a chow diet collected at ZT12 (fasted for 12 hours) were unremarkable relative to that for *2a^fl/fl^* controls (Supplemental Figure 5, A–D). These data indicate that the reduced levels of PC containing 18:2 in *L2aKO* mice is consequential to NASH diet feeding.

### Mfsd2a synergizes with the CDP/choline pathway to protect the liver from steatohepatitis.

**Figure 6:**
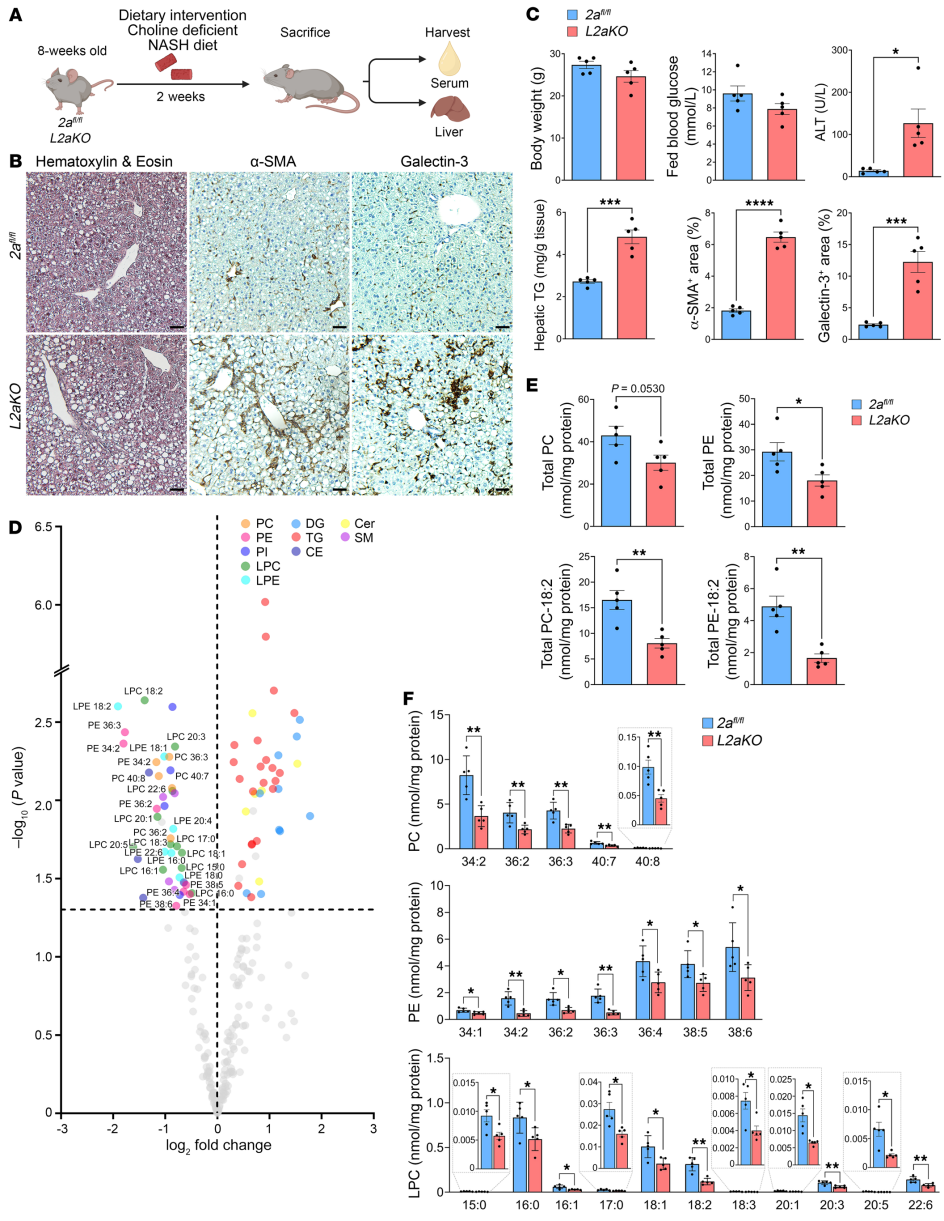
Restriction of the CDP/choline pathway exacerbates features of NASH in *L2aKO* mice. (**A**) Feeding regimen of mice fed on CD-NASH diet. Adult *2a^fl/fl^* and *L2aKO* mice were fed a CD-NASH diet for 2 weeks (*n* = 5 per genotype). (**B**) Histological analysis was performed on liver sections using H&E, α-SMA, and galectin-3. Scale bars: 50 μm. (**C**) Serum ALT and hepatic TGs were measured and morphometry analysis quantified the percentage areas that were positively stained for each of the indicated markers. (**D**) Volcano plot showing the significantly changed lipid species in *L2aKO* as compared with *2a^fl/fl^* mice (colored dots represent significant species and are located above dashed line, indicating a threshold of *P* < 0.05). (**E**) Graphs showing total PC, total PE, total PC containing 18:2, and total PE containing 18:2. (**F**) Graphs showing the specific lipid species (PC, PE, and LPC) that were significantly changed in livers of *L2aKO* as compared with *2a^fl/fl^* mice. Data are represented as means ± SEM. *P < 0.05; **P < 0.01; ***P < 0.001; ****P < 0.0001, 2-tailed Welch’s *t* test.

Mouse liver single-cell RNA-Seq data revealed that the key enzymes of the CDP/choline pathway are expressed across all zones of mouse liver, while PEMT is primarily expressed in proximity to the central vein (33), a finding also reported for PEMT in human liver (8). The overlapping expression of the CDP/choline pathway genes and Mfsd2a in periportal hepatocytes predicts that they could synergize in periportal hepatocytes to maintain hepatic PC pools and protect the liver from NAFLD. To test this concept, we attenuated the CDP/choline pathway using the classic method of feeding mice a choline-deficient/NASH diet (CD-NASH diet) (Figure 6A). As predicted, the *L2aKO* mice fed the CD-NASH diet for 2 weeks displayed more severe liver pathologies, evident from higher ALT activity, hepatic TG accumulation, inflammation, and activation of stellate cells compared with NASH diet–fed *L2aKO* mice (Figure 6, B and C). Lipidomics analysis of livers from *L2aKO* mice fed a CD-NASH diet showed an exacerbated reduction in total PC and PE relative to those fed with the NASH diet alone (Figure 5) due to reduced PC and PE species containing 18:2 fatty acid as well as cholesteryl ester (CE) containing 18:2 fatty acid (Figure 6, D and E, and Supplemental Figure 6). Minor but significant reductions in PE containing 18:1 and 3 PC/PE species containing DHA (PC-40:7, PC-40:8, and PE-38:6) were also observed in *L2aKO* livers (Figure 6F). Furthermore, LPCs that are products of PLA2 activity and are of very low abundance in liver were also significantly decreased (Figure 6F), suggestive of potential adaptations leading to sparing of PC in *L2aKO* liver. TG pools were greatly increased in *L2aKO* mice fed a CD-NASH diet, consistent with enzymatic measurements of total hepatic TG (Figure 6, C and D). Taken together, these findings support the conclusion that Mfsd2a, in parallel with the CDP/choline pathway, is critical for maintaining phospholipids containing 18:2 under HFD challenge, establishing LPC transport via Mfsd2a as a quantitatively and physiologically important pathway by which the liver maintains PC pools and protection from the development of NAFLD.

### Unsaturated LPCs promote LD biogenesis and suppress lipogenesis in Huh-7 cells.

**Figure 7:**
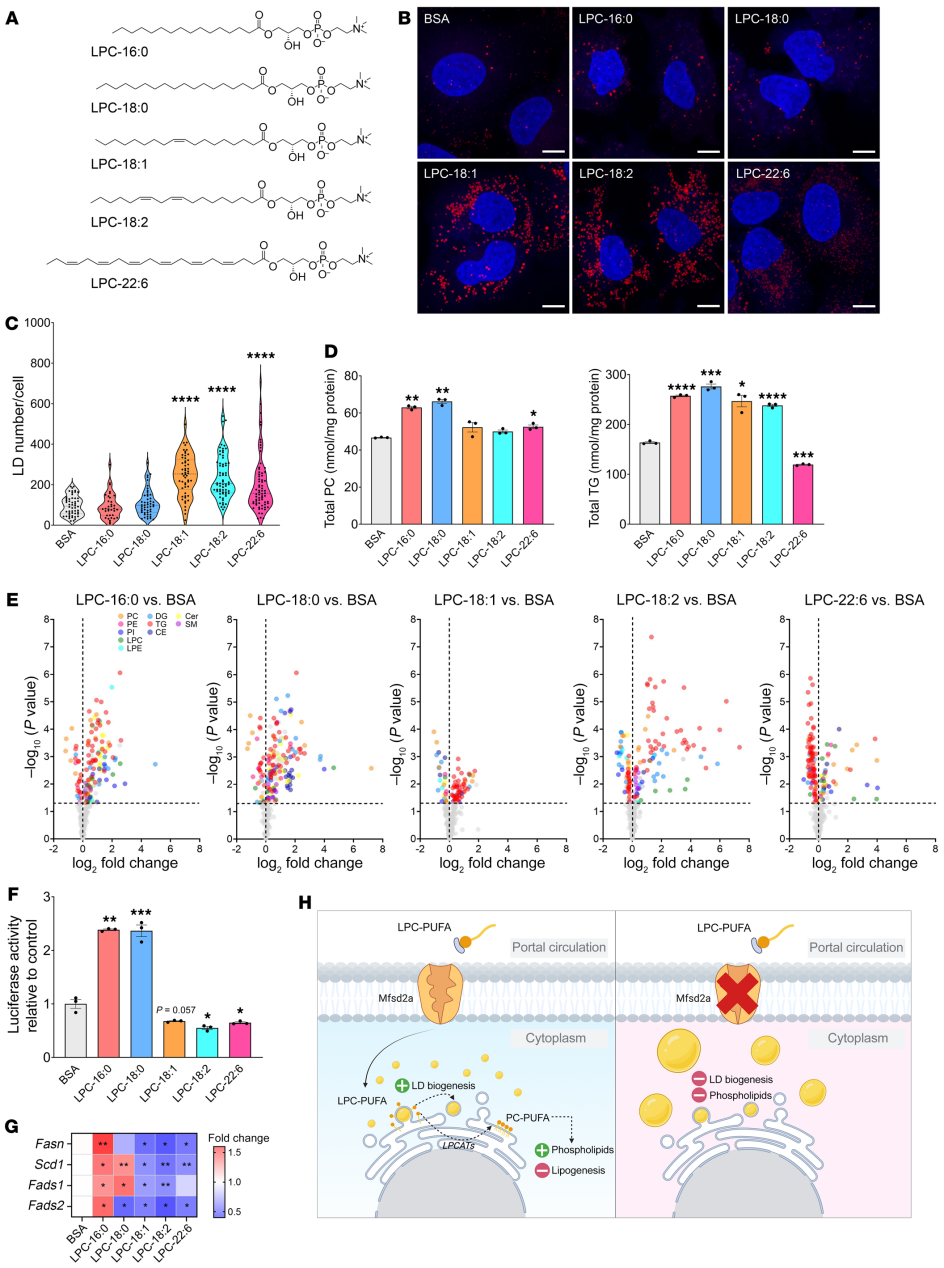
Unsaturated LPCs promote LD biogenesis and suppress lipogenesis in HuH-7 cells. (**A**) Lipid structures of LPCs used in this experiment. (**B**) Confocal microscopy of Mfsd2a-GFP–expressing HuH-7 cells treated with the indicated LPCs or fatty acid–free BSA control. LDs were stained with LipidTOX. Scale bars: 10 μm. (**C**) Violin plot shows quantification of LDs per cell. ****P < 0.0001, 2-tailed Welch’s *t* test. Each dot in the graph represents the number of LDs in a single cell treated with respective LPCs. (**D**) Lipidomic analysis of Mfsd2a-GFP–expressing HuH-7 cells treated with the indicated LPCs or BSA control (*n* = 3 technical replicates per treatment). Graphs showing total PC and TGs. (**E**) Volcano plot showing the significantly changed lipid species in cells treated with respective LPC as compared with BSA control (colored dots represent significant species and are located above dashed line, indicating a threshold of *P* < 0.05). (**F**) pSynSRE-T-Luc luciferase activity of Mfsd2a-GFP–expressing HuH-7 cells treated with respective LPCs (*n* = 3 technical replicates per treatment). Graphs showing relative luciferase activity normalized to BSA control. (**G**) Heatmap showing qPCR analysis of genes involved in lipogenesis pathway. (**H**) Proposed model of this study. Data are represented as means ± SEM. *P < 0.05; **P < 0.01; ***P < 0.001; ****P < 0.0001, 2-tailed Welch’s *t* test.

Our data indicate that LPC uptake by Mfsd2a protects the liver in part by providing LPCs for maintaining hepatic PC and PE containing 18:2. It is known that limitations in PC synthesis result in enlarged LDs (5, 39), which is a common phenotype, described histopathologically as macrosteatosis, in NAFLD (3), raising the hypothesis that specific LPCs transported by Mfsd2a might function to drive LD biogenesis. The biogenesis of LDs is considered to be hepatoprotective, acting by providing a physiological mechanism to regulate the storage and metabolism of excess dietary fatty acids and cholesterol (reviewed in ref. 40). To test this idea, we generated a human hepatoma cell line using HuH-7 cells that stably express a Mfsd2a-GFP fusion protein. HuH-7 cells do not express endogenous Mfsd2a. HuH-7 Mfsd2a-GFP cells were treated with LPCs containing either 16:0, 18:0, 18:1, 18:2, or 22:6 (Figure 7A) at 50 μM, a concentration within the physiological range for plasma LPCs (24, 41), for 8 hours under delipidated conditions, after which LDs were detected using the neutral lipid stain LipidTOX. Remarkably, LD numbers were significantly increased by the unsaturated LPCs, LPC-18:1, LPC-18:2, and LPC-22:6, but not with the saturated LPCs, LPC-16:0 and LPC-18:0 (Figure 7, A–C). Of note, LPC-18:1 and LPC-18:2 are the most abundant unsaturated LPCs in human and mouse plasma (23, 24, 41). To determine the lipid changes consequential to LPC treatment that are associated with LD formation, we subjected the cells treated with individual LPCs to lipidomic analysis. This analysis showed significantly increased levels in TGs in cells treated with all LPCs, with the exception of LPC-22:6, where cellular TG was significantly reduced relative to the control (Figure 7, D and E). Reduced TG levels in LPC-22:6–treated cells are consistent with the ability of 22:6 to suppresses DNL through inhibiting SREBP1 processing (22). Lipidomic analysis indicated that the fatty acyl chain from LPC-16:0, -18:0, -18:1, and -18:2, but not -22:6, was incorporated into TG pools (Supplemental Figure 7). In addition to contributing to TG pools, the fatty acids from LPC-16:0 and LPC-18:0 were metabolized into multiple species of PC, likely due to these LPCs being substrates for the LPC acyl transferase Lpcat3 (42, 43). Treatment of cells with the unsaturated LPC-18:1 and LPC-18:2 did not elevate total PC, but did result in significantly increased levels of PCs containing the respective 18:1 and 18:2 fatty acids (Figure 7D and Supplemental Figure 8). Performing similar experiments in cells expressing the transport-inactive Mfsd2a-GFP mutant D97A (18, 44–46) confirmed that these effects of LPCs on LD formation and the lipidome were dependent on Mfsd2a-mediated uptake of LPC into HuH-7 (Supplemental Figure 9, A and B).

It has been demonstrated that DNL is elevated in patients with NAFLD and is an important contributing factor in NAFLD (47, 48). Our RNA-Seq data from *L2aKO* mice fed with 2 weeks of the NASH diet showed upregulation of Srebp-1 target genes *Fads1* and *Fads2* (Figure 4D) relative to controls, suggesting that LPCs are regulating lipogenesis. To test this hypothesis in HuH-7 Mfsd2a-GFP cells, we quantified SREBP transcriptional activity using a classic sterol response element (SRE) luciferase reporter assay (49). Interestingly, we observed induction of SRE luciferase activity in cells treated with saturated LPC-16:0 and LPC-18:0 relative to controls. In contrast, cells treated with the unsaturated LPC-18:1, LPC-18:2, and LPC-22:6 showed a reduction in SRE luciferase activity (Figure 7F). In validation of this observation that SREBP1 activity was reduced in response to unsaturated LPC treatment, the mRNA levels of the lipogenic Srebp-1 target genes *Fasn*, *Scd1*, *Fads1*, and *Fads2* were significantly reduced in cells treated with unsaturated LPCs (Figure 7G). These findings support the conclusion that unsaturated LPCs both drive LD formation and repress DNL.

## Discussion

Phospholipids play an important role in safeguarding the liver from the pathogenic consequences of dietary lipids, and it is thought that dysregulation of phospholipid metabolism is involved in the pathogenesis of NAFLD (reviewed in ref. 4). Whether pathways for the exogenous uptake of phospholipid other than through lipoprotein uptake by hepatocytes exist has not been explored. In this study, we discovered that the expression of the LPC transporter Mfsd2a in the liver is under the control of GR action to respond to dietary fat feeding, where it plays an essential role in protecting the liver from NAFLD in the presence of HFD challenge.

Mfsd2a was originally identified as a fasting-induced orphan transporter in mouse liver (28, 29). Similar to mouse liver, human liver is one of the top Mfsd2a-expressing organs (50). Despite this knowledge, a physiological role for the uptake of blood-derived LPCs into hepatocytes has remained enigmatic. *Mfsd2a* is under circadian control, where peak expression occurs at the onset of mealtime (30). The circadian control of *Mfsd2a* via the SCN is consistent with our finding that *Mfsd2a* is a direct target of GR and coincides with peak GC levels prior to mealtime. Interestingly, GR and PPARα are noncore clock components under circadian control that show enhanced circadian peak activity in overnutrition models (51, 52), explaining the induction of Mfsd2a expression during the early stages of HFD challenge shown in the current study. The dual regulation of Mfsd2a expression by PPARα and GR suggests important roles in regulating hepatic uptake of LPC for preparing the liver for dietary fat intake.

Our study demonstrated that *Mfsd2a* mRNA and protein levels are elevated in NASH patients, hinting at a role of Mfsd2a in limiting NAFLD progression. The rapidity and severity of disease onset in livers of *L2aKO* mice fed a NASH diet is notable. *L2aKO* mice fed a NASH diet or CD-NASH diet recapitulated many of the histopathological findings of human NASH, namely macrosteatosis, portal fibrosis, lobular inflammation, and immune cell infiltration. These findings are uncommon in mouse models in which dietary-induced NASH is slow, taking 12 to 32 weeks to reach NAFLD with mild NASH that does not progress to NASH with advanced fibrosis. The fact that *L2aKO* exhibited macrosteatosis, activation of HSC, and inflammation after only 2 weeks of challenge with a NASH diet indicates a critical role for LPC transport in hepatoprotection during overnutrition. It is interesting to consider that upregulation of Mfsd2a in periportal hepatocytes in response to dietary challenge limits periportal pathology in humans, in which pericentral pathology is the norm in adults with NAFLD and NASH (53, 54). It is appreciated that periportal disease is associated with more adverse outcomes relative to pericentral disease, particularly with regard to fibrosis (53, 54).

The spatial and temporal expression of Mfsd2a at mealtime points to a specific role for LPC transport in portal hepatocytes. Based on our data, we propose that Mfsd2a transports LPC–polyunsaturated fatty acids (LPC-PUFAs) prior to mealtime (ZT12) to prepare the liver for feeding. During HFD feeding, dietary lipid-rich blood containing chylomicron remnants and fatty acids enters the liver through portal circulation that would be anticipated to cause an increase in demand of PC lipids for the packaging of neutral lipids into LDs. Moreover, oxygenated blood from hepatic arteries located at the portal zone creates an oxygen-rich environment where fatty acid oxidation genes under the control of PPARα are expressed that could contribute to reactive oxygen species and lipid peroxidation with increasing dietary fat intake (55–57). Indeed, periportal hepatocytes are more susceptible to ischemia/reperfusion injury than pericentral hepatocytes (58). Of note, PC containing 18:2 is prone to lipid peroxidation and forms highly reactive PC-containing oxidized phospholipids (OxPLs) (reviewed in ref. 59). OxPLs have been shown to accumulate in multiple murine NASH models and patients with NASH (60), where evidence suggests that OxPLs contributes to hyperinflammation (61). Furthermore, stable isotope–tracer studies in mice have estimated that turnover of newly synthesized PC in the liver is on the order of 50% of total liver PC per hour (6), indicating that the liver has an enormous physiological demand for replacing and sustaining PC pools. Under conditions of overnutrition leading to the development of NAFLD and NASH, the Mfsd2a pathway for uptake of plasma-derived LPCs likely becomes rate limiting for maintaining liver PC levels, a conclusion supported by our lipidomic data showing reduced 18:2 in PC pools, particularly when PC synthesis via the CDP/choline pathway is reduced. These findings suggest that one of the important physiological functions of plasma LPC-18:2 is to replenish hepatic PC-18:2 pools.

We show that, in addition to serving as a source for hepatic PC, transport of unsaturated LPCs into hepatocytes promotes LD biogenesis. Interestingly, unlike the phospholipid composition of the rough ER, unsaturated LPC is abundant on the LD phospholipid shell (13), suggesting that accumulation of LPC at specific sites at the ER might be important for LD biogenesis. Our study here demonstrates that unsaturated LPCs induced both LD formation and suppressed DNL (i.e., fatty acid synthesis), seemingly contradicting processes that can be reconciled by the observation that the fatty acid moiety in the LPCs added to cells was partially metabolized into TG pools, thus elevating cellular TG and LD formation. An important exception to this observation was the fact that LPC-22:6 was preferentially metabolized into PC and induced LD formation while reducing cellular TGs. These combined findings indicate that LD formation can be independent of lipogenesis and TG biosynthesis. Studies from model systems have shown that LPCs can induce LD formation. A recent example of this comes from an in vitro study that demonstrated that the conical shape of LPC favored LD budding in giant unilamellar vesicles (GUVs) (62). Moreover, conversion of PC to LPC on GUVs using phospholipase A2 treatment decreased surface tension and facilitated droplet budding (62). A cell-based study using the budding yeast *Saccharomyces cerevisiae* has also shown that unsaturated LPCs can induce LD formation (63). These studies have speculated that the source of LPC for LD formation is derived from endogenous cellular phospholipase activity, but this remains unproven. Our study provides evidence that an important physiological source of LPC for both LD formation and maintenance of hepatic PC pools is blood derived and taken up by hepatocytes via Mfsd2a.

In summary, our findings reveal that uptake of blood-derived LPCs mediated by Mfsd2a constitutes a GR-regulated pathway by which the liver obtains LPCs for maintaining hepatic PCs and that is essential for liver health in the face of overnutrition. Our study shows that the hepatoprotective effects attributed to the Mfsd2a/LPC pathway are 3-fold: (a) LPC-18:1 and LPC-PUFA induce LD formation; (b) LPCs replenish hepatic PC pools; and (c) LPC-PUFA reduces DNL (Figure 7H). Indeed, reduction of DNL is considered a therapeutic modality for treating NAFLD (45, 64, 65). Of relevance to NAFLD, these findings suggest that reductions in hepatic Mfsd2a expression or blood levels of LPC-18:1 and LPC-PUFA could contribute to susceptibility for the development of this disease. Moreover, this study raises the possibility that a dietary source of LPC containing PUFA could be hepatoprotective.

## Methods

### Animals.

All mice were housed on a 12-hour light/12-hour dark cycle with a humidity and temperature-controlled environment at 23°C. The mice were fed ad libitum with standard normal chow diet (SF00-100, Specialty Feeds) and given free access to water. For the circadian experiments, mice were entrained for 7 days on a 12-hour light/12-hour dark cycle. Diet was removed at ZT0 and replenished at ZT12, and the process was repeated for 7 consecutive days. The mouse model used in this study is as described in Supplemental Methods.

Only male mice were used in this study unless otherwise stated. Eight-week-old adult mice were placed on a NASH diet (composed of 41% fat, 17% protein, 43% carbohydrate; D12079Bi, Research Diet Inc.) or CD-NASH diet (identical to NASH diet except without choline; D11042101i, Research Diet Inc.), and supplemented with 15% w/v fructose (F0127, Sigma-Aldrich) drinking water for the indicated feeding regimens.

All mice were sacrificed at ZT4 to avoid the confounding effect of circadian rhythms. Animals were anesthetized with 200 mg/kg ketamine and 20 mg/kg xylazine in PBS, followed by blood collection via cardiac puncture. Mice were then perfused with PBS before the liver was extracted and snap-frozen in liquid nitrogen or fixed in buffered formaldehyde solution (ICM Pharma). Serum was collected by allowing the blood to clot at room temperature for 30 minutes, and blood was centrifuged at 1,500*g*, 4°C for 10 minutes.

### Blood parameters.

Fed blood glucose was measured with an Accu-Chek glucometer (Roche) through tail-vein bleeding. Serum ALT was measured using ALT/SGPT Liqui-UV (2930-430, Stanbio Laboratory).

### Quantification of hepatic TGs.

Liver lipid isolations were performed as described (66). In brief, frozen livers were homogenized in 20 volume (v/w) of chloroform/ethanol (2:1 v/v) with glass beads, and 0.2 volume of 0.9% saline was added to the homogenate and shaken vigorously at room temperature for 1 hour. Lysate was centrifuged at 400*g* for 10 minutes. The organic phase was dried under a stream of nitrogen gas and resuspended in 1% Triton X-100. The hepatic TGs and cholesterol were measured with the Triglyceride LiquiColor Mono Kit (2200-225, Stanbio Laboratory).

### Hydroxyproline quantification.

Liver hydroxyproline was quantified using the Colorimetric Hydroxyproline Assay Kit (ab222941, Abcam). In brief, frozen liver was homogenized in nuclease-free water (10 ml water/mg tissue) using a MagNA Lyser instrument (Roche), and 100 ml of 10M NaOH was added to 100 ml of liver homogenates and incubated at 120°C for 1 hour. The hydrolysates were then cooled down on ice, neutralized with 100 ml concentrated 10M HCl, and centrifuged at 10,000*g* for 5 minutes. Supernatants were then subjected to hydroxyproline quantification according to the manufacturer’s instructions.

### Histology.

Histological procedures are as described in Supplemental Methods.

### Western blot analysis.

An enriched liver membrane fraction was isolated. In brief, 100 mg of frozen liver was Dounce homogenized in 20 volumes of hypotonic buffer (v/w) (10 mM HEPES, pH 7.50, 10 mM MgCl_2_, 10 mM KCl, 5 mM NaCl) supplemented with protease inhibitor cocktail (11836170001, Roche). Homogenates were incubated on ice for 30 minutes. Next, lysates were passed 30 times though a 23-gauge needle. Lysates were then spun at 500*g* for 10 minutes. The cleared supernatant was centrifuged at 100,000*g* at 4°C for 30 minutes. The membrane pellet was then resuspended in high-salt buffer (10 mM HEPES, pH 7.50, 10 mM MgCl_2_ 10 mM KCl, 1M NaCl) supplemented with protease inhibitor cocktail (Roche). The suspension was centrifuged at 100,000*g* at 4°C for 30 minutes. The membrane pellet was resuspended in solubilization buffer (20 mM HEPES, pH 7.50, 200 mM NaCl, 1% Fos-choline 13; F310, Anatrace) supplemented with protease inhibitor cocktail (Roche) and incubated overnight at 4°C.

Protein concentration was determined using the BCA Protein Assay Kit (23227, Thermo Scientific). Lysates for each sample were mixed with 5× SDS loading buffer, respectively, to a final concentration of ×1 (30 mM Tris-HCl at pH 7.4, 3% w/v SDS, 5% v/v glycerol, 0.004% w/v bromophenol blue,and 2.5% v/v β-mercaptoethanol) (67) and resolved by SDS-PAGE. Proteins were transferred to nitrocellulose membrane (Bio-Rad) and blocked with 5% blotting grade blocker (1706404, Bio-Rad). Immunodetection was performed as described (22).

### RNA isolation.

Total RNA was isolated by homogenizing mouse liver in TRIzol reagent (15596026, Invitrogen) using a MagNA Lyser instrument (Roche) and purified using the RNeasy Mini Kit (74106, QIAGEN) according to the manufacturer’s recommended protocol. The concentration of RNA was determined using Nanodrop (Thermo Fisher Scientific).

### Quantitative PCR.

Liver cDNA was synthesized from 1 μg of liver total RNA using iScript Reverse Transcriptase Supermix (170-8841, Bio-Rad) according to the manufacturer’s recommended protocol. Quantitative PCR (qPCR) was performed with the SensiFAST SYBR Hi-ROX Kit (BIO-92005, Bioline) using the CFX384 Touch Real-Time PCR Detection System (Bio-Rad). qPCR primers used in this study are listed in Supplemental Data Set 1.

### RNA-Seq and analysis.

Human bulk liver RNA-Seq from normal-weight patients, obese patients, patients with NAFLD, and patients with NASH was obtained from the NCBI’s Gene Expression Omnibus database (GEO GSE126848). RNA-Seq analysis was performed with Partek Flow (version 10). The RNA-Seq reads were mapped against hg19 using the STAR-2.7.8a aligner. Differential expression analysis was performed using DESeq2.

For mouse RNA-Seq analysis, liver RNA library preparation and RNA-Seq were performed by NovogeneAIT (Singapore). RNA-Seq analysis was performed with Partek Flow (version 10). The RNA-Seq reads were mapped against mm10 using the STAR-2.7.8a aligner. Differential expression analysis was performed using DESeq2, and a gene was considered significantly differentially expressed with a *P* value of less than 0.05 and a fold change of 1.25 or greater.

### Cell culture.

HeLa and HEK293T cells were obtained from ATCC. Cells were maintained in DMEM (high-glucose DMEM containing 100 units/mL penicillin and 100 μg/mL streptomycin) supplemented with 10% FBS. HuH-7 cells (JCRB0403) were obtained from the RIKEN BRC Cell Bank and maintained in low-glucose DMEM containing 100 units/ml penicillin and 100 μg/mL streptomycin and supplemented with 10% FBS. All cells were cultured in 5% CO_2_ at 37°C.

### Luciferase assay.

HeLa cells were seeded onto 12-well tissue culture dishes (1.5 × 10^5^ cells/well) and cultured in DMEM supplemented with 10% FBS. The next day, cells were cotransfected with 500 ng of firefly luciferase constructs with 5 ng of renilla luciferase using Lipofectamine 2000. After transfection, the medium was replaced with DMEM supplemented with 5% charcoal-stripped FBS and treated with 100 nM Dex or vehicle control (DMSO), respectively, for 24 hours. For HuH-7 cells, 0.5 × 10^5^ cells/well were seeded in 12-well plates and cultured in low-glucose DMEM supplemented with 10% FBS. The next day, cells were transfected with 1 mg pSynSRE-T-Luc (Addgene, 60444) and 50 ng of renilla luciferase using Lipofectamine 3000. After 5 hours incubation, cells were washed twice with serum-free DMEM and medium supplemented with 5% delipidated FBS was added. On day 2, cells were treated with BSA-bound LPCs in DMEM supplemented with 5% delipidated FBS for 8 hours. Cell luciferase activity was determined with the Dual-Luciferase Reporter Assay System (Promega, E1960) according to the manufacturer’s recommendations. Luminescence measurements were made with a Tecan Spark 10M microplate reader. Relative luciferase activity was expressed as the ratio of firefly luminescence to renilla luminescence normalized to control.

### Generation of HuH-7 cells expressing Mfsd2a-GFP.

The HuH-7 expressing Mfsd2a-GFP stable cell line was established by lentiviral transduction. Lentivirus particles were produced by transfecting HEK293T cells with FUGW-Mfsd2a-GFP or FUGW-Mfsd2a (D97A)-GFP, pMDLG/PRRE (Addgene, 12251), pRSV-Rev (Addgene, 12253), and pCMV-VSV-G (Addgene, 8454) plasmids (at a 2:2:2:1 plasmid ratio) with polyethylenimine (Sigma-Aldrich, 408727). Media containing lentiviral particles were collected at 48 hours and filtered with a 0.45 μM membrane filter. Polybrene (Sigma Aldrich, 107689) was added to a final concentration of 8 μg/ml into the medium containing the lentiviral particles and used to transduce HuH-7 cells. HuH-7 cells expressing Mfsd2a-GFP were isolated by FACS.

### LPC treatment of HuH-7 Mfsd2a-GFP cells.

On day 0, HuH-7 Mfsd2a-GFP cells were seeded onto 8-well glass chamber slides (0.5 × 10^4^ cells/well) for LD droplet imaging or in 6-well tissue culture dishes (1.5 × 10^4^ cells/well) for lipidomics and cultured in media as described above supplemented with 10% FBS. The following day, cells were washed twice with serum-free DMEM and medium supplemented with 5% delipidated FBS was added. On day 2, cells were treated with BSA-bound LPCs in DMEM supplemented with 5% delipidated FBS for 8 hours.

### LD staining and microscopy.

Cells were fixed with 3% electron microcopy–grade paraformaldehyde for 10 minutes and washed twice with PBS. LDs were stained with HCS LipidTOX Neutral Lipid Stain (Thermo Scientific, H34476) at 1:500 dilution in PBS for 2 hours. Cells were washed twice with PBS, and nuclei were counterstained for 10 minutes with Hoechst 33342 (Thermo Scientific, H3570) at a dilution of 1:500. Images were captured using the laser scanning confocal inverted microscope LSM710 (Carl Zeiss) with a Plan-Apochromat ×63/1.40 oil DIC M27 lens, and 14-optical *Z*-sections at 1 μm intervals were captured. Images are maximal projections of the *Z*-stacks. LD quantification was performed using the Find Maxima plugin in ImageJ (NIH).

### Lipid extraction of cells.

Cells were washed twice with 0.5% fatty acid–free BSA in PBS and once with PBS. Lipid was extracted from cells twice with 0.5 ml hexane:isopropanol (3:2, v/v) for 30 minutes. The combined lipid extracts were then dried under a stream of argon gas. Following lipid extraction and air drying of cells to remove the remaining liquid, cell lysis buffer (0.1% SDS, 0.1M NaOH) was added to the cells on the plate for 16 hours at 4°C. Protein concentration from solubilized cells was determined with the BCA Protein Assay Kit.

### Preparation of hepatic lipid homogenates for lipidomic analysis.

Frozen livers were homogenized in 20-volume homogenization buffer (v/w) (150 mM ammonium bicarbonate with glass beads). Protein concentration of the homogenate was measured using the BCA Protein Assay Kit.

### Lipidomics.

Lipidomics analysis was performed as described in Supplemental Methods.

### Graphical illustrations and figures.

Graphical illustrations were created with BioRender. Figures and schematic diagrams were generated with Adobe Photoshop 2022. Graphs were plotted with Graphpad Prism 9.

### Statistics.

All samples were biological replicates and are presented as mean ± SEM. Two-tailed, unpaired Welch’s *t* test and 1-way ANOVA with Dunnett’s or Tukey’s test were employed to determine the significance of the differences between the means of experimental groups. *P* < 0.05 was considered to be significant. All statistical tests were performed with GraphPad Prism 9 (GraphPad Software).

### Study approval.

Collection and use of human liver biopsies for IHC analysis was approved by SingHealth CIRB (protocol number CIRB ref 2021/2046). All mouse procedures were performed in accordance with and approved by the SingHealth Institutional Animal Care and Use Committee (IACUC protocol number 2015/SHS/1416).

### Data availability.

RNA-Seq data were deposited in the NCBI’s Gene Expression Omnibus database (GEO GSE173545). Values for all data points in graphs are reported in the Supporting Data Values file.

## Author contributions

CFC and DLS conceived of and designed the experiments. CFC, DLAG, LG, BHW, BCT, GLC, RYJL, MSL, WQL, and GBBG performed the experiments. CFC, BHW, LG, YCL, MRW, MSL, WQL, GBBG, FT, and DLS analyzed the data. CFC and DLS wrote the paper.

## Supplementary Material

Supplemental data

Supplemental data set 1

Supporting data values

## Version 1 07/18/2023

In-Press Preview

## Version 2 09/01/2023

Electronic publication

## References

[1] Ipsen DH (2018). Molecular mechanisms of hepatic lipid accumulation in non-alcoholic fatty liver disease. Cell Mol Life Sci.

[2] Listenberger LL (2003). Triglyceride accumulation protects against fatty acid-induced lipotoxicity. Proc Natl Acad Sci U S A.

[3] Tandra S (2011). Presence and significance of microvesicular steatosis in nonalcoholic fatty liver disease. J Hepatol.

[4] van der Veen JN (2017). The critical role of phosphatidylcholine and phosphatidylethanolamine metabolism in health and disease. Biochim Biophys Acta Biomembr.

[5] Krahmer N (2011). Phosphatidylcholine synthesis for lipid droplet expansion is mediated by localized activation of CTP:phosphocholine cytidylyltransferase. Cell Metab.

[6] Pynn CJ (2011). Specificity and rate of human and mouse liver and plasma phosphatidylcholine synthesis analyzed in vivo. J Lipid Res.

[7] Hall Z (2017). Lipid zonation and phospholipid remodeling in nonalcoholic fatty liver disease. Hepatology.

[8] Wattacheril J (2013). Differential intrahepatic phospholipid zonation in simple steatosis and nonalcoholic steatohepatitis. PLoS One.

[9] Gyorgy P, Goldblatt H (1942). Observations on the conditions of dietary hepatic injury (necrosis, cirrhosis) in rats. J Exp Med.

[10] Lyman RL (1973). Phosphatidylethanolamine metabolism in rats fed a low methionine, choline-deficient diet. Lipids.

[11] Weltman MD (1996). Increased hepatocyte CYP2E1 expression in a rat nutritional model of hepatic steatosis with inflammation. Gastroenterology.

[12] Bartz R (2007). Lipidomics reveals that adiposomes store ether lipids and mediate phospholipid traffic. J Lipid Res.

[13] Tauchi-Sato K (2002). The surface of lipid droplets is a phospholipid monolayer with a unique fatty acid composition. J Biol Chem.

[14] Vance JE (2015). Phospholipid synthesis and transport in mammalian cells. Traffic.

[15] Kennedy EP, Weiss SB (1956). The function of cytidine coenzymes in the biosynthesis of phospholipides. J Biol Chem.

[16] DeLong CJ (1999). Molecular distinction of phosphatidylcholine synthesis between the CDP-choline pathway and phosphatidylethanolamine methylation pathway. J Biol Chem.

[17] Bremer J, Greenberg DM (1961). Methyl transfering enzyme system of microsomes in the biosynthesis of lecithin (phosphatidylcholine). Biochim Biophys Acta.

[18] Nguyen LN (2014). Mfsd2a is a transporter for the essential omega-3 fatty acid docosahexaenoic acid. Nature.

[19] Wong BH (2016). Mfsd2a Is a Transporter for the essential ω-3 fatty acid docosahexaenoic acid (DHA) in eye and is important for photoreceptor cell development. J Biol Chem.

[20] Lobanova ES (2019). Disrupted blood-retina lysophosphatidylcholine transport impairs photoreceptor health but not visual signal transduction. J Neurosci.

[21] Croset M (2000). Characterization of plasma unsaturated lysophosphatidylcholines in human and rat. Biochem J.

[22] Chan JP (2018). The lysolipid transporter Mfsd2a regulates lipogenesis in the developing brain. PLoS Biol.

[23] Alakbarzade V (2015). A partially inactivating mutation in the sodium-dependent lysophosphatidylcholine transporter MFSD2A causes a non-lethal microcephaly syndrome. Nat Genet.

[24] Guemez-Gamboa A (2015). Inactivating mutations in MFSD2A, required for omega-3 fatty acid transport in brain, cause a lethal microcephaly syndrome. Nat Genet.

[25] Scala M (2020). Biallelic MFSD2A variants associated with congenital microcephaly, developmental delay, and recognizable neuroimaging features. Eur J Hum Genet.

[26] Harel T (2018). Homozygous mutation in MFSD2A, encoding a lysolipid transporter for docosahexanoic acid, is associated with microcephaly and hypomyelination. Neurogenetics.

[27] Pu W (2016). Mfsd2a+ hepatocytes repopulate the liver during injury and regeneration. Nat Commun.

[28] Berger JH (2012). Major facilitator superfamily domain-containing protein 2a (MFSD2A) has roles in body growth, motor function, and lipid metabolism. PLoS One.

[29] Angers M (2008). Mfsd2a encodes a novel major facilitator superfamily domain-containing protein highly induced in brown adipose tissue during fasting and adaptive thermogenesis. Biochem J.

[30] Hughes ME (2012). Brain-specific rescue of Clock reveals system-driven transcriptional rhythms in peripheral tissue. PLoS Genet.

[31] Mae MA (2021). Single-cell analysis of blood-brain barrier response to pericyte loss. Circ Res.

[32] MacParland SA (2018). Single cell RNA sequencing of human liver reveals distinct intrahepatic macrophage populations. Nat Commun.

[33] Halpern KB (2017). Single-cell spatial reconstruction reveals global division of labour in the mammalian liver. Nature.

[34] Suppli MP (2019). Hepatic transcriptome signatures in patients with varying degrees of nonalcoholic fatty liver disease compared with healthy normal-weight individuals. Am J Physiol Gastrointest Liver Physiol.

[35] Grontved L (2013). C/EBP maintains chromatin accessibility in liver and facilitates glucocorticoid receptor recruitment to steroid response elements. EMBO J.

[36] Castro-Mondragon JA (2022). JASPAR 2022: the 9th release of the open-access database of transcription factor binding profiles. Nucleic Acids Res.

[37] Kadiyala V (2016). Cistrome-based cooperation between airway epithelial glucocorticoid receptor and NF-κB orchestrates anti-inflammatory effects. J Biol Chem.

[38] Li Z (2006). The ratio of phosphatidylcholine to phosphatidylethanolamine influences membrane integrity and steatohepatitis. Cell Metab.

[39] Fei W (2011). A role for phosphatidic acid in the formation of “supersized” lipid droplets. PLoS Genet.

[40] Gluchowski NL (2017). Lipid droplets and liver disease: from basic biology to clinical implications. Nat Rev Gastroenterol Hepatol.

[41] Croset M (2000). Characterization of plasma unsaturated lysophosphatidylcholines in human and rat. Biochem J.

[42] Rong X (2015). Lpcat3-dependent production of arachidonoyl phospholipids is a key determinant of triglyceride secretion. Elife.

[43] Hishikawa D (2008). Discovery of a lysophospholipid acyltransferase family essential for membrane asymmetry and diversity. Proc Natl Acad Sci U S A.

[44] Cater RJ (2021). Structural basis of omega-3 fatty acid transport across the blood-brain barrier. Nature.

[45] Quek DQ (2016). Structural insights into the transport mechanism of the human sodium-dependent lysophosphatidylcholine transporter MFSD2A. J Biol Chem.

[46] Chua GL (2023). Mfsd2a utilizes a flippase mechanism to mediate omega-3 fatty acid lysolipid transport. Proc Natl Acad Sci U S A.

[47] Lambert JE (2014). Increased de novo lipogenesis is a distinct characteristic of individuals with nonalcoholic fatty liver disease. Gastroenterology.

[48] Kim CW (2017). Acetyl CoA Carboxylase inhibition reduces hepatic steatosis but elevates plasma triglycerides in mice and humans: a bedside to bench investigation. Cell Metab.

[49] Dooley KA (1998). Sterol regulation of 3-hydroxy-3-methylglutaryl-coenzyme A synthase gene through a direct interaction between sterol regulatory element binding protein and the trimeric CCAAT-binding factor/nuclear factor Y. J Biol Chem.

[50] GTEx Consortium (2013). The genotype-tissue expression (GTEx) project. Nat Genet.

[51] Quagliarini F (2019). Cistromic reprogramming of the diurnal glucocorticoid hormone response by high-fat diet. Mol Cell.

[52] Guan D (2018). Diet-induced circadian enhancer remodeling synchronizes opposing hepatic lipid metabolic processes. Cell.

[53] Rakha EA (2010). Portal inflammation is associated with advanced histological changes in alcoholic and non-alcoholic fatty liver disease. J Clin Pathol.

[54] Brunt EM (2009). Portal chronic inflammation in nonalcoholic fatty liver disease (NAFLD): a histologic marker of advanced NAFLD-Clinicopathologic correlations from the nonalcoholic steatohepatitis clinical research network. Hepatology.

[55] Freitas I (2016). In Situ evaluation of oxidative stress in rat fatty liver induced by a methionine- and choline-deficient diet. Oxid Med Cell Longev.

[56] Jungermann K, Kietzmann T (1997). Role of oxygen in the zonation of carbohydrate metabolism and gene expression in liver. Kidney Int.

[57] Sato B (1995). Quantitative analysis of redox gradient within the rat liver acini by fluorescence images: effects of glucagon perfusion. Biochim Biophys Acta.

[58] Taniai H (2004). Susceptibility of murine periportal hepatocytes to hypoxia-reoxygenation: role for NO and Kupffer cell-derived oxidants. Hepatology.

[59] Binder CJ (2016). Innate sensing of oxidation-specific epitopes in health and disease. Nat Rev Immunol.

[60] Sun X (2020). Neutralization of oxidized phospholipids ameliorates non-alcoholic steatohepatitis. Cell Metab.

[61] Di Gioia M (2020). Endogenous oxidized phospholipids reprogram cellular metabolism and boost hyperinflammation. Nat Immunol.

[62] Ben M’barek K (2017). ER membrane phospholipids and surface tension control cellular lipid droplet formation. Dev Cell.

[63] Choudhary V (2018). Architecture of lipid droplets in endoplasmic reticulum is determined by phospholipid intrinsic curvature. Curr Biol.

[64] Calle RA (2021). ACC inhibitor alone or co-administered with a DGAT2 inhibitor in patients with non-alcoholic fatty liver disease: two parallel, placebo-controlled, randomized phase 2a trials. Nat Med.

[65] Stiede K (2017). Acetyl-coenzyme A carboxylase inhibition reduces de novo lipogenesis in overweight male subjects: A randomized, double-blind, crossover study. Hepatology.

[66] Folch J (1957). A simple method for the isolation and purification of total lipides from animal tissues. J Biol Chem.

[67] Hannah VC (2001). Unsaturated fatty acids down-regulate srebp isoforms 1a and 1c by two mechanisms in HEK-293 cells. J Biol Chem.

