# Supplementary material for: Blood-derived lysophospholipid sustains hepatic phospholipids and fat storage necessary for hepatoprotection in overnutrition
Source: J Clin Invest. 2023 Sep 1;133(17):e171267. doi: 10.1172/JCI171267 (PMC10471173; doi:10.1172/JCI171267)
Supplement: Supplemental data [file jci-133-171267-s135.pdf]

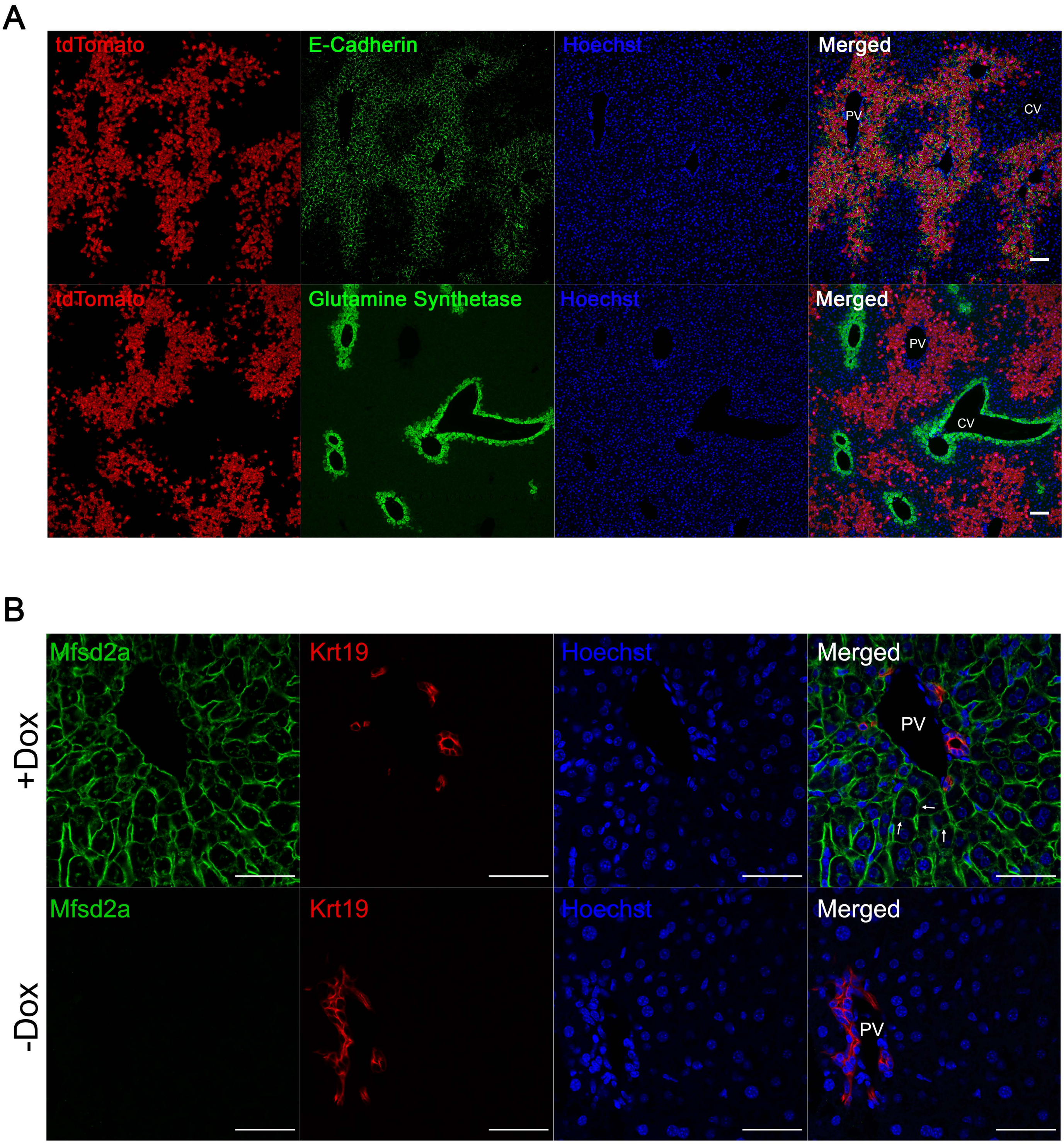

Supplemental Figure 1. Spatial regulation of hepatic Mfsd2a expression. (A) Immunostaining of tdTomato at the periportal region in liver sections from *Mfsd2a-ERT2Cre Rosa26-tdTomato* mouse. E-cadherin and Glutamine synthetase are periportal and pericentral hepatocyte markers, respectively (n = 3 mice). Scale bar: 100µm. (B) Immunostaining of Mfsd2a in liver section from *Alb-Cre Rosa26-rtTA-Mfsd2a* mouse fed a doxycycline containing normal chow and normal chow control respectively. Krt19, a cholangiocyte marker, indicated the portal triad. White arrows indicate bile canaliculi (n=3 mice per treatment). PV: portal vein, CV: central vein. Scale bar: 50 µm.

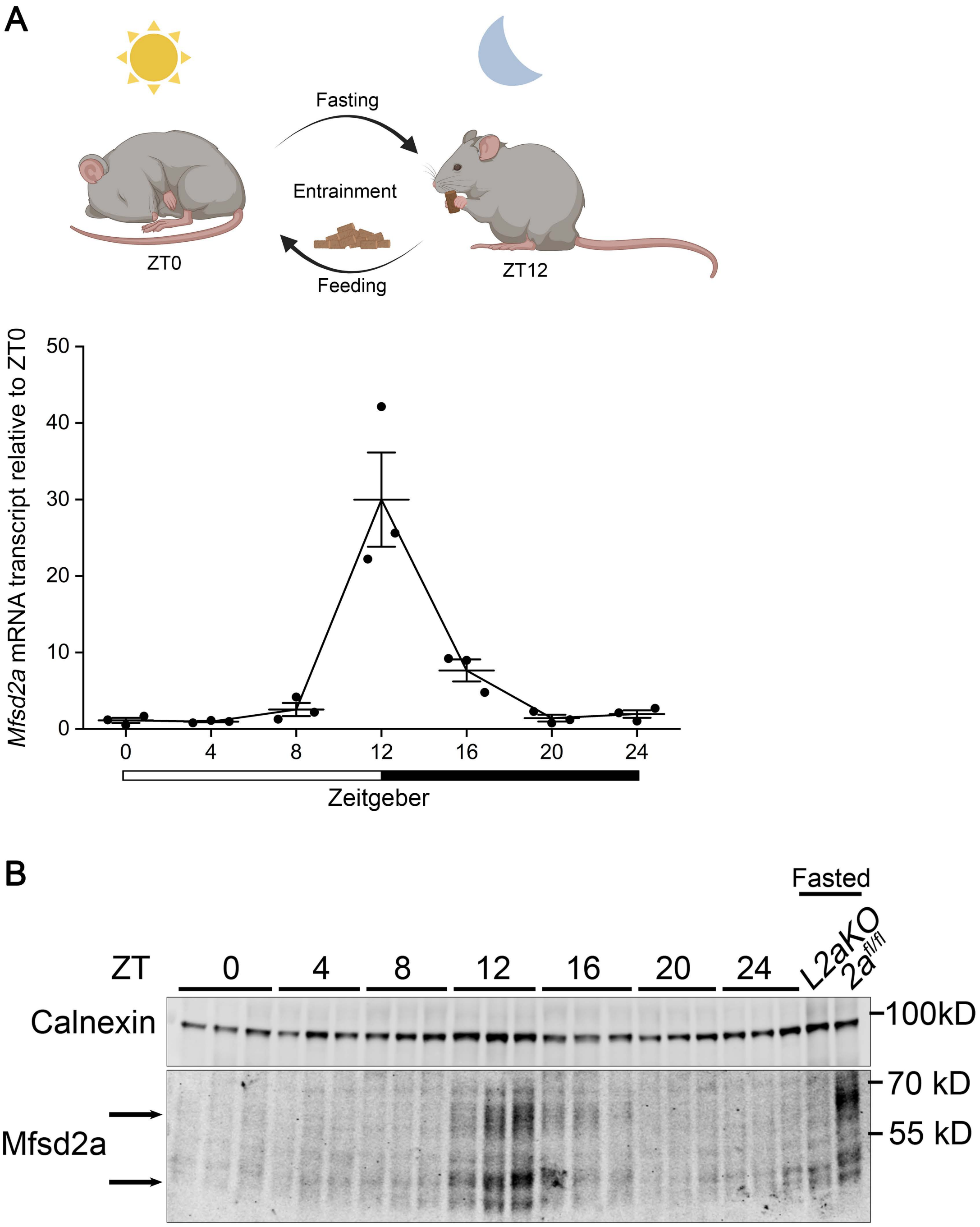

Supplemental Figure 2. Circadian control of *Mfsd2a* expression. (A) Circadian expression of *Mfsd2a* mRNA analyzed by qPCR throughout a 24 hr circadian period. ZT0 is the lights-on time and ZT12 is the lights-off time. Data are represented as *Mfsd2a* mRNA normalized to β-actin and relative to average expression at ZT0 ± SEM (n=3 mice per genotype). (B) Immunoblot showing the protein expression of Mfsd2a at respective ZT. Calnexin was used as a loading control.

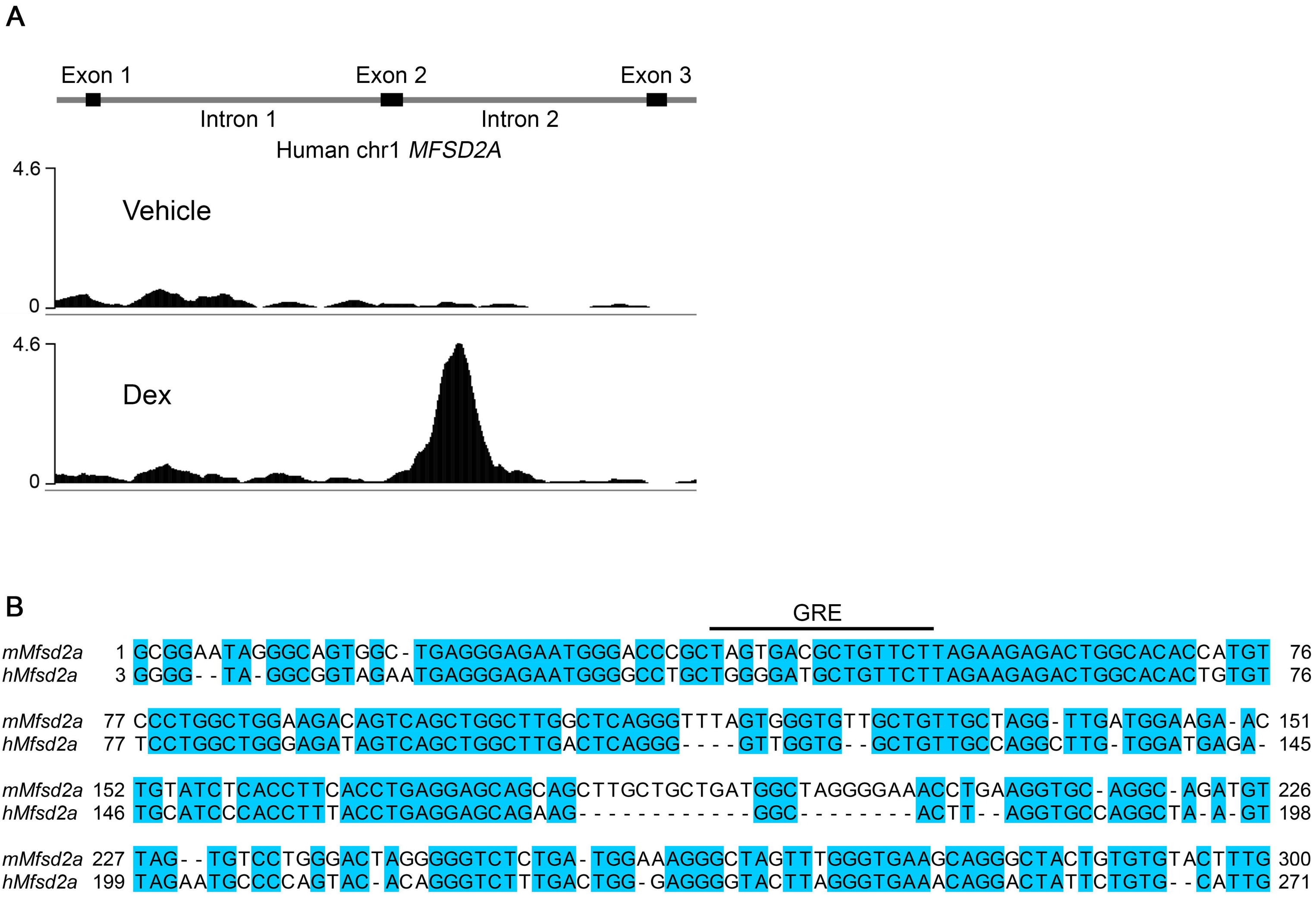

Supplemental Figure 3. GR occupancy at *Mfsd2a* intron 2 is conserved between mouse and human. (A) A ChIP-seq profile showing GR occupancy at intron 2 of human *MFSD2A* in Dex but not vehicle treated BEAS-2B cells. (B) Pairwise sequence alignment of DNA region encompassing the GR peak at *Mfsd2a* intron 2 in mouse and human has 72% sequence similarity. Black horizontal line shows the GRE at mouse *Mfsd2a* intron 2 identified in this study.

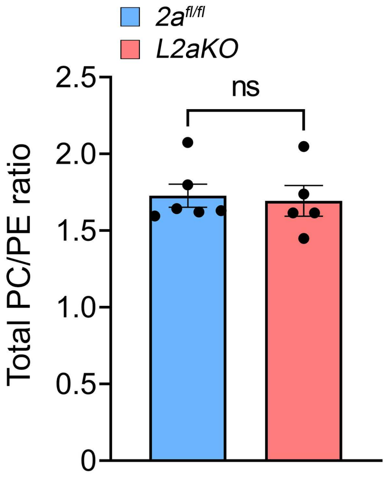

Supplemental Figure 4. Lipidomic analysis for total PC to PE ratio. Graph showing total PC/PE ratio in livers of *2a*$^{fl/fl}$ (n=6) and *L2aKO* mice (n=5) fed with 2-weeks NASH diet. Data are represented as mean ± SEM. NS indicates not statistically significant, by two-tailed Welch's t-test.

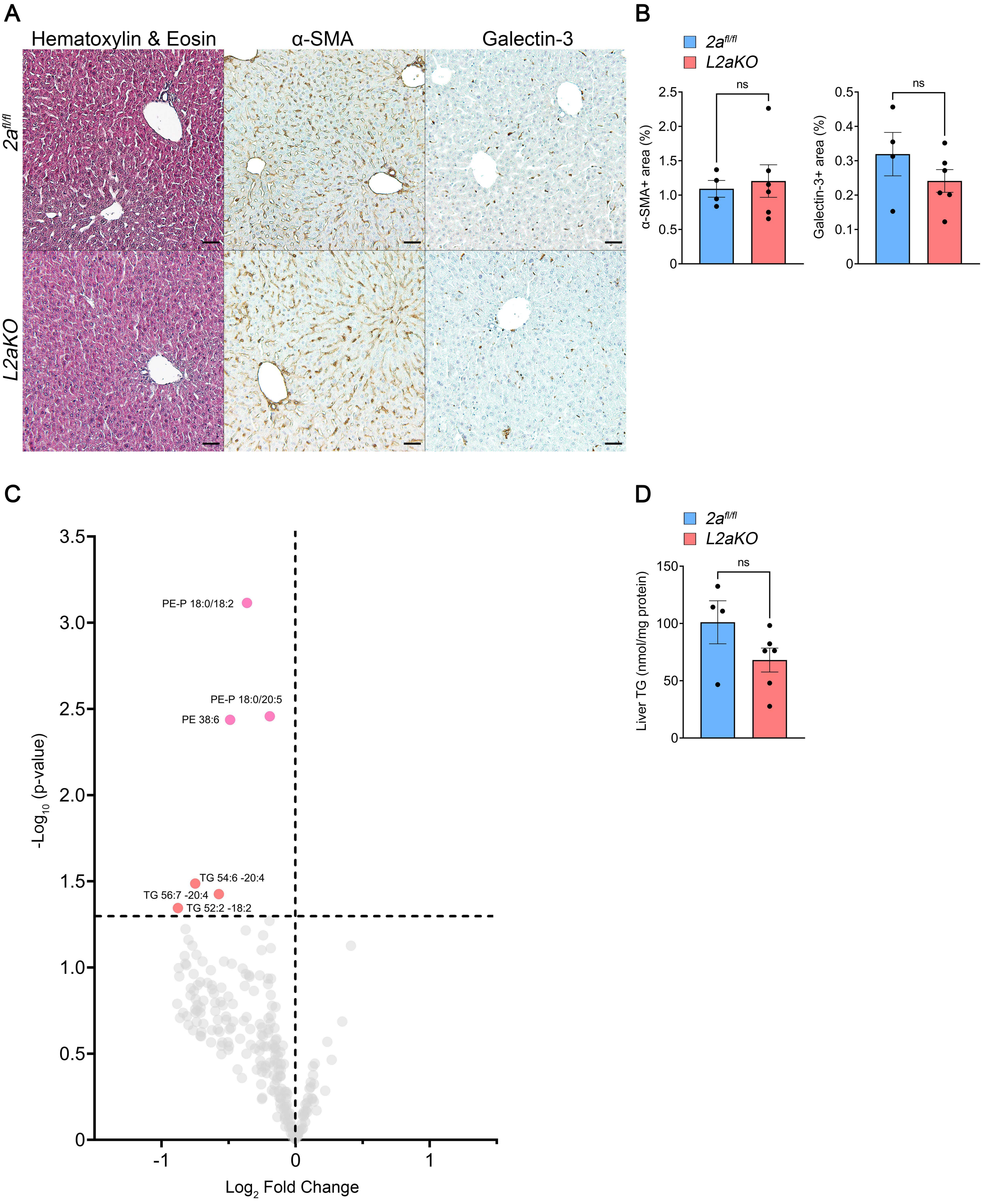

Supplemental Figure 5. Analysis of *L2aKO* livers from mice fed normal chow diet. Circadian entrained adult *2a*$^{fl/fl}$ (n=4) and *L2aKO* (n=6) mice were fed with normal chow. Livers were harvested at ZT12 (fasted for 12 hours). (A) Histological analysis was performed on liver sections using H&E, α-SMA, and, Galectin-3. Scale bar = 50μm. (B) Morphometry analysis quantified the percent area that were positively stained for each of the indicated markers. (C) Volcano plot showing the significantly changed lipid species in *L2aKO* as compared to *2a*$^{fl/fl}$ mice (colored dots are significant species and above dashed line indicating a threshold of $p<0.05$). (D) Graph showing total hepatic TG from *2a*$^{fl/fl}$ and *L2aKO* mice fed with normal chow. Data are represented as mean ± SEM. Statistical analysis was performed by two-tailed Welch's t-test.

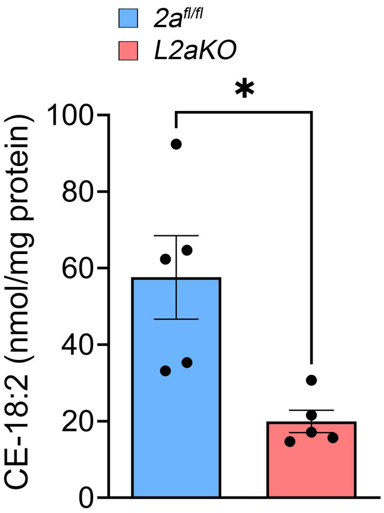

Supplemental Figure 6. Lipidomic analysis of cholesteryl esters. Graph showing cholesteryl ester-18:2 in liver from *2a*$^{fl/fl}$ and *L2aKO* mice fed with 2-weeks CD-NASH diet (n=5 per genotype). Data are represented as mean ± SEM, *p<0.05, by two-tailed Welch's t-test.

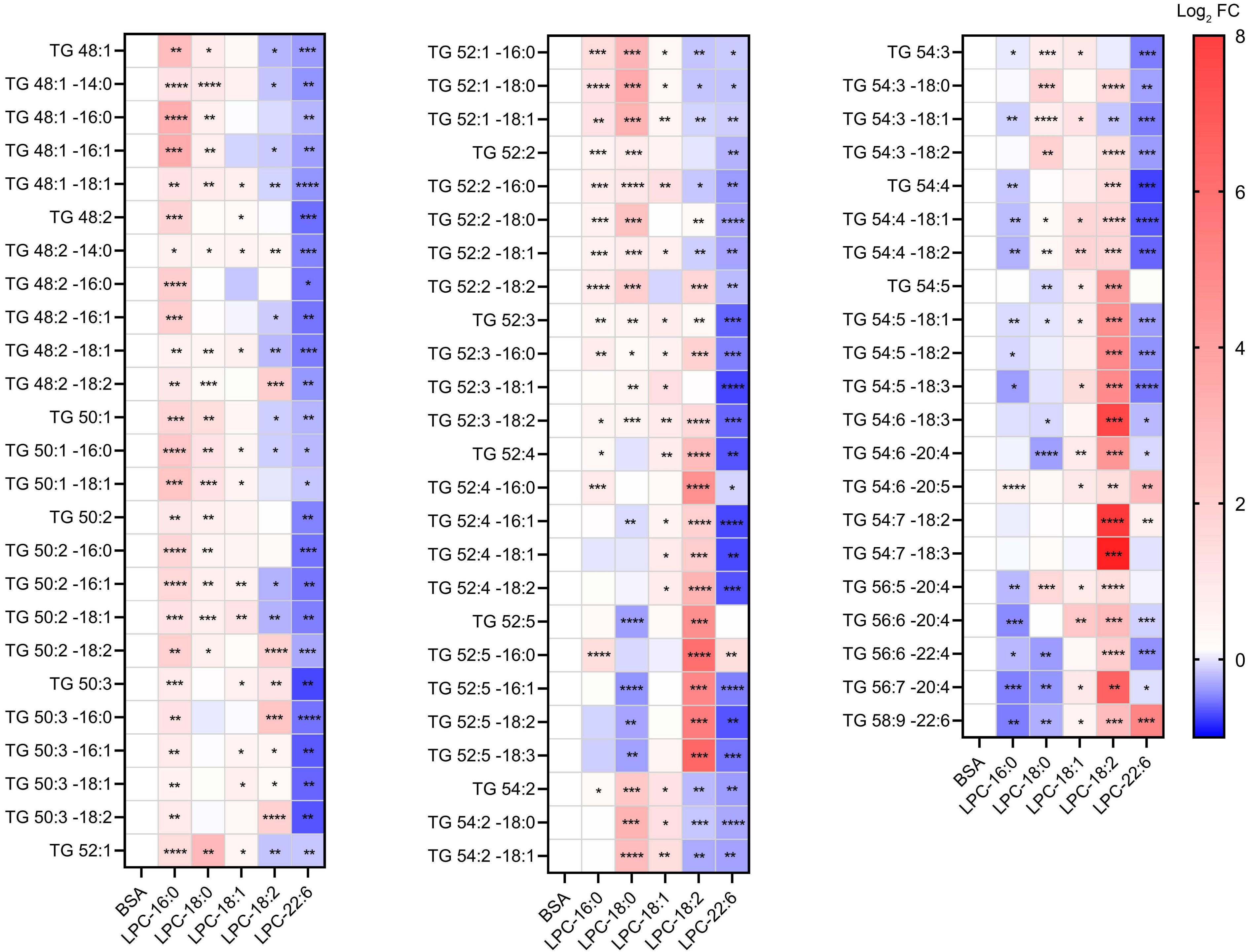

Supplemental Figure 7. Effects of LPC treatment on TG species in HuH-7 Mfsd2a-GFP cells. Heatmap showing TG species quantified from lipidomic analysis of cells treated with indicated LPCs. Data are expressed as log2 fold change (FC) of lipid with indicated LPC treated cells relative to BSA treated control cells, *p<0.05, **p<0.01, ***p< 0.001, and ****p< 0.0001 by two-tailed Welch's t-test.

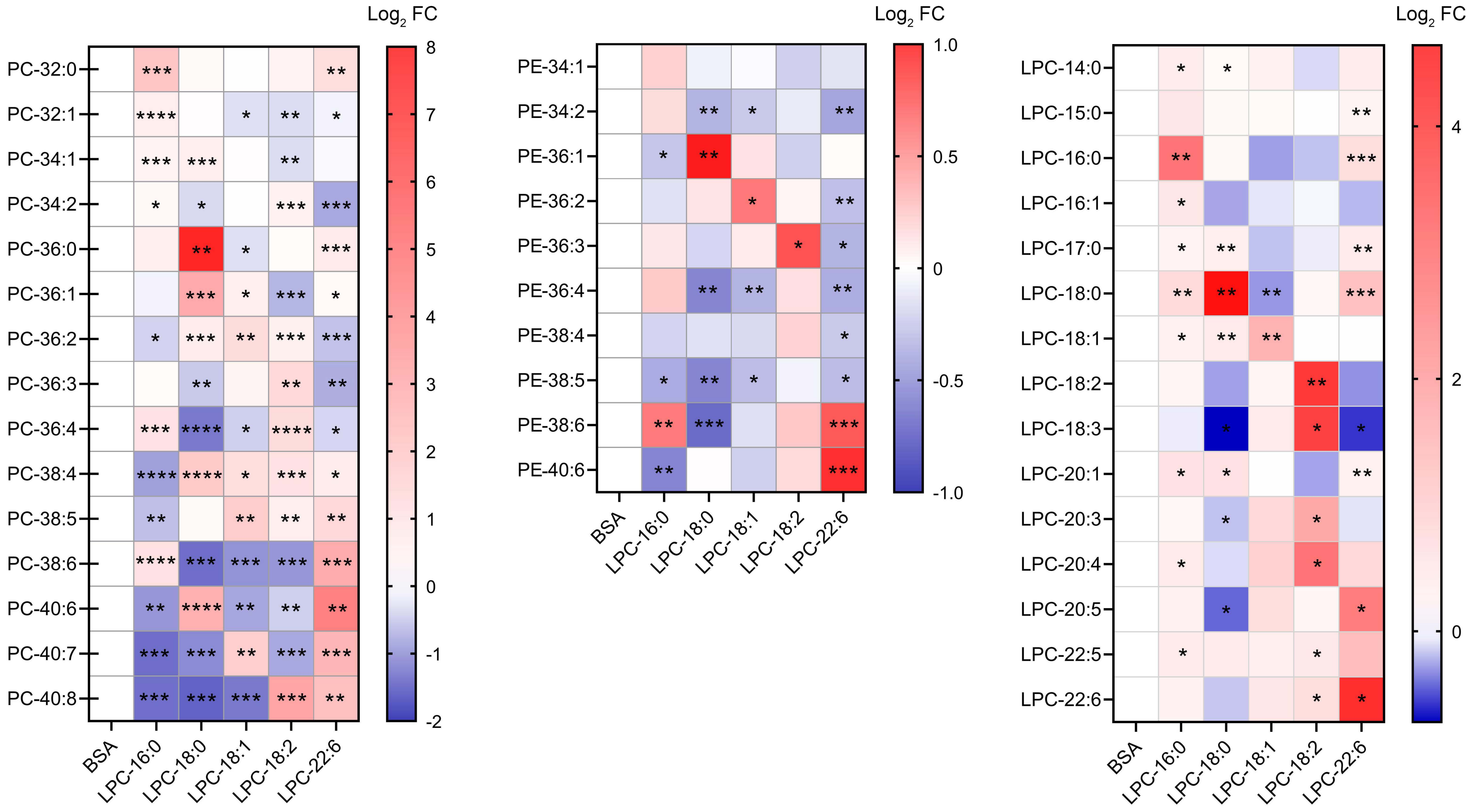

Supplemental Figure 8. Effects of LPC treatment on phospholipid species in HuH-7 Mfsd2a-GFP cells. Heatmap showing phospholipids species quantified from lipidomic analysis of cells treated with indicted LPCs. Data are expressed as $\log_2$ fold change (FC) of lipid with indicated LPC treated cells relative to BSA treated control cells, *p<0.05, **p<0.01, ***p< 0.001, and ****p< 0.0001 by two-tailed Welch's t-test.

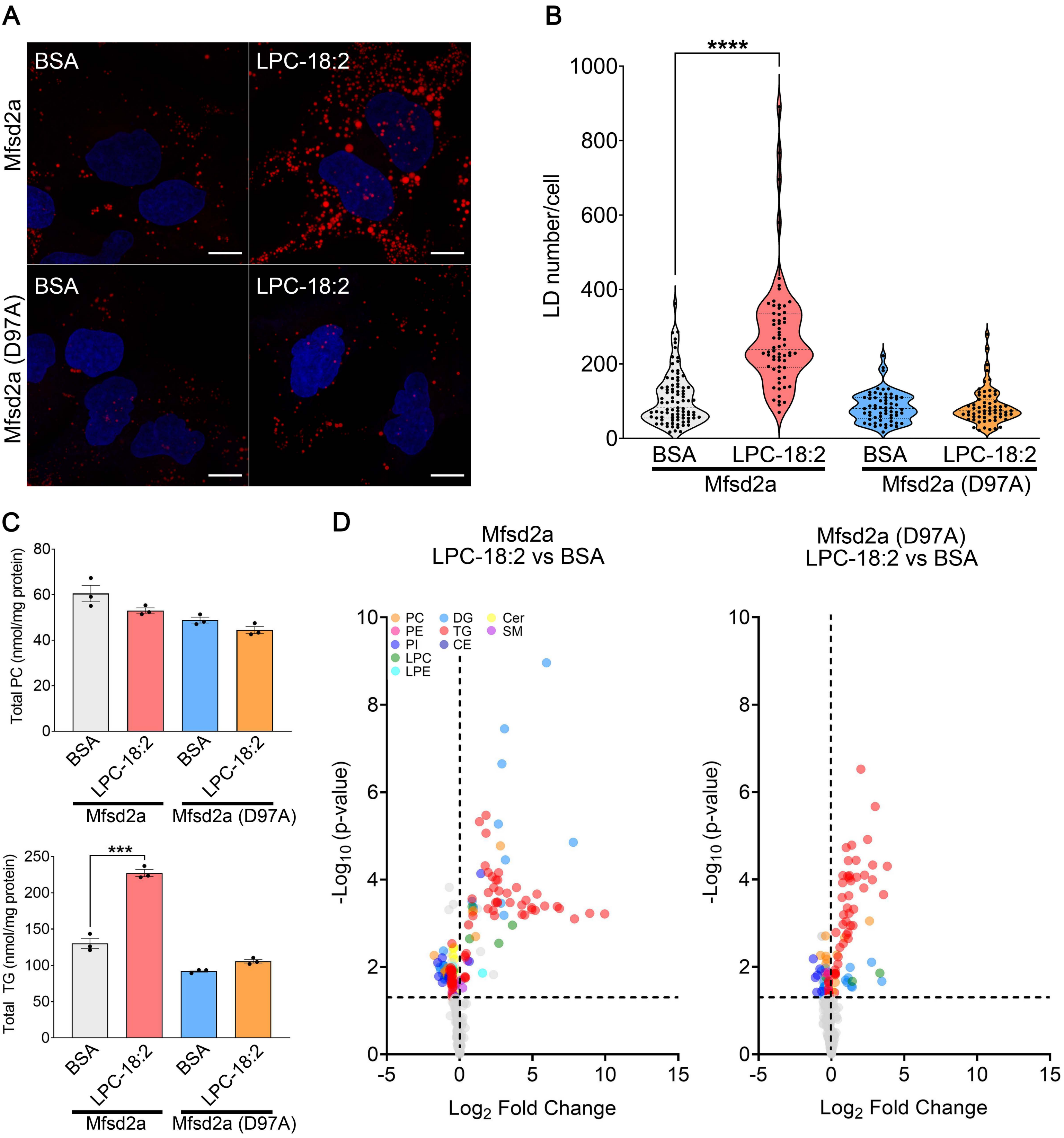

Supplemental Figure 9. Effects of LPC on LD formation and lipidome are Mfsd2a-dependent. (A) Confocal microscopy of HuH-7 cells expressing Mfsd2a-GFP or Mfsd2a (D97A)-GFP treated with 50μM LPC-18:2 or BSA control. LDs and cell nuclei were stained LipidTox and Hoechst 33342, respectively. Scale bar = 10μm. (B) Violin plot shows quantification of LD per cell. Asterisk denotes p-value, ****$p<0.0001$, by two-tailed Welch's t-test. Each dot in the graph represent the number of LD in a single cell treated with LPC-18:2 or BSA control. (C) Lipidomics analysis of Mfsd2a-GFP or Mfsd2a (D97A)-GFP expressing HuH-7 cells treated with 50μM LPC-18:2 or BSA control (n=3 technical replicates, per treatment). Graphs show total PC and total TG. Data are represented as mean ± SEM, ***$p<0.001$, by two-tailed Welch's t-test. (D) Volcano plot shows the significantly changed lipid species in Mfsd2a-GFP or Mfsd2a (D97A)-GFP expressing HuH-7 cells treated with 50μM LPC-18:2 or BSA control (colored dots are significant species and located above dashed line indicating a threshold of $p<0.05$).

No.	Subject Number	Gender	Age	Type of Fatty Liver (NASH/Non-NASH)	Steatosis Grade	Lobular Inflammation Grade	Ballooning Grade	Fibrosis Stage
1	M001	M	28	NASH	3	2	1	1a
2	M002	M	56	NASH	1	3	1	3
3	M003	F	74	NASH	1	2	1	3
4	M004	F	67	NASH	2	1	1	1a
5	M005	F	61	NASH	2	2	0	3
6	M006	F	43	Non-NASH	3	0	0	0
7	M007	M	40	Non-NASH	1	0	0	0
8	M008	F	52	Non-NASH	1	2	0	3
9	M009	F	49	Non-NASH	2	1	0	0
10	M010	M	65	Non-NASH	1	0	0	1

Supplemental Table 1. Histological assessment of liver biopsies from patients with NASH (n=5) and without NASH (n=5).

Supplemental Methods

Mouse Model

Mfsd2a-CreERT2 Rosa26-tdtomato mice were generated by crossing Mfsd2a specific tamoxifen inducible Cre to *ROSA26-tdTomato* reporter mice (1). To induce recombination, 200mg/kg body weight tamoxifen (T5648, Sigma-Aldrich) was dissolved in corn oil and administered via intraperitoneal injection for two consecutive days in female mice.

GR floxed mice (2) were obtained from The Jackson Laboratory. Liver-specific deletion of GR (*LGRKO*) were generated by crossing *Alb-Cre*$^{+/-}$ (3) to *GR*$^{fl/fl}$ mice. *Mfsd2a* floxed mice (*2a*$^{fl/fl}$) were generated as previously described (4). Liver-specific deletion of Mfsd2a (*L2aKO*) mice were generated by crossing *Alb-Cre*$^{+/-}$ (3) to *2a*$^{fl/fl}$ mice.

Liver specific tetracycline-inducible Mfsd2a mice were generated by crossing the *Alb-Cre*$^{+/-}$ line to a mouse line harboring CAGGS promoter upstream of a loxp-flanked termination sequence that is followed by a rtTA coding sequence and a bovine growth hormone terminator (bGHpA) at the *Rosa26* locus. A tetO sequence was inserted downstream of bGHpA to induce mouse Mfsd2a transgene expression in the presence of tetracycline bound rtTA.

Histology

Livers were fixed in buffered formaldehyde solution (PFA) (ICM Pharma) overnight at room temperature. For paraffin sections following overnight PFA fixation, livers were transferred to 70% ethanol overnight followed by embedding in paraffin. Livers were cut at 5μm thickness with a microtome (Leica Biosystems) and stained with H&E, or Sirius red, or subjected to immunohistochemistry analysis (IHC).

Immunohistochemistry

For detecting α-SMA (ab32575, abcam) paraffin embedded liver sections were deparaffinized, rehydrated, and subjected to antigen retrieval in microwave-boiled citrate buffer (10mM citric acid, pH6.0) for 1 hour. For detecting Galectin-3 (125402, Biolegend) liver sections were treated as above but instead of citrate buffer treatment, sections were incubated for 10 minutes in Proteinase K solution (20μg/ml Proteinase K, BIO-37084, Bioline in 50mM Tris, 1mM EDTA, pH8.0). Sections were then rinsed with water and endogenous peroxidase activity was inhibited with BLOXALL blocking solution (SP-6000, Vector Laboratories) for 30 minutes. Next, sections were washed 3 times with TBS-T (50mM Tris-HCl pH7.40, 150mM NaCl, 0.1% Tween-20), blocked with blocking buffer (5% normal goat serum; 10000C, Invitrogen in TBS-T) and incubated in primary antibody diluted in blocking buffer overnight at 4°C. Anti-Galectin-3, and anti-α-SMA were used at 1:400 dilution. After three washes with TBS-T, sections were incubated with horseradish peroxidase conjugated secondary antibody (Vector Laboratories) for 1 hour. Sections were washed 3 times with TBS-T and subjected to

3,3'-Diaminobenzidine (DAB) (SK-4105, Vector Laboratories) staining. The nuclei were counterstained with hematoxylin before mounting with CV ultra-mounting media (14070936261, Leica Biosystems). Images were captured using a light microscope (Olympus) with the 10x objective.

Histology Quantification

Percent area with IHC stained cells were quantified with ImageJ (NIH). Three fields (10x objective) per section from each mouse liver were subjected for analysis.

Immunofluorescence

Paraffin-embedded livers sections were dewaxed and rehydrated, followed by antigen retrieval using Tris-EDTA buffer (10mM tris base, 1mM EDTA, pH9.0) for E-cadherin staining or citrate buffer (10mM citric acid, pH6.0) for Glutamine synthetase staining. After antigen retrieval, sections were permeabilization with 0.1% Triton X-100 in PBS (137mM NaCl, 2.7mM KCl, 10mM Na_2HPO_4, 1.8mM KH_2PO_4) for 10min and incubated with PBS-TX blocking buffer (5% normal goat serum, 0.01% Triton X-100 in PBS). Liver sections were incubated with respective primary antibody diluted in PBS-TX overnight at 4^oC. Anti-RFP (600-401-379, Rockland) and anti-E-Cadherin (610182, BD Biosciences) were used at 1:250, anti-Glutamine Synthetase (MAB302, Millipore) was used at 1:500 dilution. After three washes with PBS-TX, sections were incubated with Alexa Fluor Plus secondary antibody diluted in PBS-TX (1:250) for 1 hour. Sections were washed three times with PBS-TX, and nuclei were stained with Hoechst 33342 for 5 min, and

mounted with Fluorsave Reagent (345789, Merck). Images were captured using a Leica Fluorescence microscope (Leica Biosystems).

Lipidomics

10µL of homogenized tissue were mixed with 490µL of butanol:methanol (1:1, v/v) spiked with internal standards (IS). The standards used were acylcarnitine 16:0 D3, cholesterol ester 18:0 D6, ceramide d18:0/08:0, ceramide d18:1/12:0, deoxyceramide m18:1/12:0, cholesterol D7, diacylglycerol 15:0/15:0, GM3 ganglioside d18:1/18:0 D3, monohexosylceramide d18:1/12:0, dihexosylceramide d18:1/12:0, trihexosylceramide d18:1/18:0 D3, lysophosphatidylcholine 13:0, lysophosphatidylethanolamine 14:0, phosphatidylcholine 13:0/13:0, plasmalogen phosphatidylcholine 18:0/18:1 D9, phosphatidylethanolamine 17:0/17:0, plasmalogen phosphatidylethanolamine 18:0/18:1 D9, phosphatidylglycerol 17:0/17:0, phosphatidylinositol 12:0/13:0, phosphatidylserine 17:0/17:0, sphingomyelin d18:1/12:0, sphinganine d17:0, sphingosine d17:1, triacylglycerol 12:0/12:0/12:0 and were purchased from Avanti Lipids. The mixture was vortexed for 2 min, sonicated for 30 min and then centrifuged twice at 4°C (14,000 g for 10 min). The supernatant fraction was collected for LC-MS/MS analysis. A pooled lipid extract was used as a quality control (QC) sample and injected every 5 study samples. The LC-MS/MS analysis was performed on an Agilent UHPLC 1290 Infinity II liquid chromatography system connected to an Agilent QqQ 6495C.

Liquid chromatography for the RPLC separation was carried out using an Agilent Zorbax RRHD Eclipse Plus C18 column (2.1 × 50 mm, 1.8 μm). The mobile phases A (60% water and 40% acetonitrile with 10 mmol/L ammonium formate) and B (10% acetonitrile and 90% isopropanol with 10 mmol/L ammonium formate) were used for the chromatographic separation. The following gradient was applied: 0-2 min, 20-60% B; 2-12 min, 60-100% B; 12-14 min, 100% B; 14.01-15.8 min, 20% B. The oven temperature was maintained at 40°C. Flow rate was set at 0.4 mL/min and the sample injection volume was 1 μL.

For QqQ: The positive ionization spray voltage and nozzle voltage were set at 3,000 V and 1,000 V, respectively. The drying gas and sheath gas temperatures were both maintained at 250°C. The drying gas and sheath gas flow rates were 14 L/min and 11 L/min, respectively. The nebulizer nitrogen gas flow rate was set at 35 psi. The iFunnel high and low pressure RF were 150 V and 60 V, respectively. Targeted analysis was performed in Dynamic MRM positive ion mode. The acquired MS data were analyzed using Agilent MassHunter software version B.08.00.

For normalization and quantification: The signal to noise ratios (S/N) were calculated using the raw peak areas in study samples and processed blanks (PBLK). Lipids that had S/N < 10, CV > 20% in the QC samples and did not show a linear behaviour (R^2 < 0.8) in dilution curves were excluded from the analysis. Internal standards were used to normalize the raw peak areas in the corresponding lipid class and concentrations were

further normalized to the protein concentration in the original sample. Due to the methodology used in our study, we are not able to report absolute concentration values. Endogenous species were quantified using one standard per lipid class thus our method can only deliver relative quantitation results.

References

1. Madisen L, Zwingman TA, Sunkin SM, Oh SW, Zariwala HA, Gu H, Ng LL, Palmiter RD, Hawrylycz MJ, Jones AR, et al. A robust and high-throughput Cre reporting and characterization system for the whole mouse brain. *Nat Neurosci.* 2010;13(1):133-40.

2. Mittelstadt PR, Monteiro JP, and Ashwell JD. Thymocyte responsiveness to endogenous glucocorticoids is required for immunological fitness. *J Clin Invest.* 2012;122(7):2384-94.

3. Postic C, Shiota M, Niswender KD, Jetton TL, Chen Y, Moates JM, Shelton KD, Lindner J, Cherrington AD, and Magnuson MA. Dual roles for glucokinase in glucose homeostasis as determined by liver and pancreatic beta cell-specific gene knock-outs using Cre recombinase. *J Biol Chem.* 1999;274(1):305-15.

4. Wong BH, Chan JP, Cazenave-Gassiot A, Poh RW, Foo JC, Galam DL, Ghosh S, Nguyen LN, Barathi VA, Yeo SW, et al. Mfsd2a Is a Transporter for the Essential omega-3 Fatty Acid Docosahexaenoic Acid (DHA) in Eye and Is Important for Photoreceptor Cell Development. *J Biol Chem.* 2016;291(20):10501-14.
